# Disfunção Persistente do Ventrículo Direito e Evolução Hemodinâmica Predizem Deterioração Hospitalar na Embolia Pulmonar de Risco Intermediário-Alto e Alto

**DOI:** 10.36660/abc.20260468

**Published:** 2026-07-31

**Authors:** Miguel A. Quintana, Joaquín Perea, Ricardo Villarreal, Mauricio Soto, Marildes Castro, Rafael Cortês, Patricio Sanhueza, Iván Saavedra, Yhaquelina Quispe, Roger Tenezaca, Roberto Colque, Alexandra Arias, Alex Rivera Toquica, Manuel Álvarez, Ysmenia D. Pérez, Paola Morejón, Karina G. Fernández, Adriana Mogollón, Marcelo Cardona, Gonzalo Emanuel Pérez, Arnaldo López-Ruiz

**Affiliations:** 1 Cardiopulomonary Institute LW Randall Asunción Paraguai Cardiopulomonary Institute LW Randall – Cardiovascular, Asunción – Paraguai; 2 Sanatorio Guemes Unidad Coronaria Buenos Aires Argentina Sanatorio Guemes – Unidad Coronaria, Buenos Aires – Argentina; 3 Hospital Hernán Henríquez Aravena Temuco Chile Hospital Hernán Henríquez Aravena – Cardiología, Temuco – Chile; 4 Faculdade de Medicina Universidade Federal de Minas Gerais Belo Horizonte MG Brasil Faculdade de Medicina – Universidade Federal de Minas Gerais – Cardiologia, Belo Horizonte, MG – Brasil; 5 Hospital Sírio - Libanês Brasília Brasília DF Brasil Hospital Sírio - Libanês Brasília – Cardiologia, Brasília, DF – Brasil; 6 Universidad de Chile Santiago Chile Universidad de Chile – Cardiologia, Santiago – Chile; 7 Biocor Hospital de Doenças Cardiovasculares Ltda Belo Horizonte MG Brasil Biocor Hospital de Doenças Cardiovasculares Ltda – Cardiologia, Belo Horizonte, MG – Brasil; 8 Hospital Obrero No 1 La Paz Bolívia Hospital Obrero No 1, La Paz – Bolívia; 9 Hospital de especialidades Guayaquil Abel Gilbert Ponton Guayaquil Equador Hospital de especialidades Guayaquil Abel Gilbert Ponton, Guayaquil – Equador; 10 Sanatorio Allende Córdoba Argentina Sanatorio Allende – Cardiologia, Córdoba – Argentina; 11 Instituto Nacional de Cardiología Ignacio Chávez State of Mexico México Instituto Nacional de Cardiología Ignacio Chávez, State of Mexico – México; 12 Clinica los Rosales Risaralda Colômbia Clinica los Rosales, Risaralda – Colômbia; 13 Fundacion Santa Fe de Bogota Bogotá Colômbia Fundacion Santa Fe de Bogota – Cardiologia, Bogotá – Colômbia; 14 Hospital General y Especialidades Dr.Mario Tolentino Dipp Santo Domingo República Dominicana Hospital General y Especialidades Dr.Mario Tolentino Dipp – Cardiologia, Santo Domingo – República Dominicana; 15 Clinica Guayaquil Guayaquil Equador Clinica Guayaquil – Cardiologia, Guayaquil – Equador; 16 Hospital de Emergencias José Casimiro Ulloa Lima Peru Hospital de Emergencias José Casimiro Ulloa – Cardiologia, Lima – Peru; 17 Centro Medico San Francisco Caracas Venezuela Centro Medico San Francisco – Cardiologia, Caracas – Venezuela; 18 Hola Hospital Privado de Rosario Rosario Argentina Hola Hospital Privado de Rosario – Cardiologia, Rosario – Argentina; 19 Clinica Olivos Buenos Aires Argentina Clinica Olivos – Cardiology, Buenos Aires – Argentina; 20 AdventHealth Orlando Department of Critical Care Orlando Florida EUA AdventHealth Orlando – Department of Critical Care, Orlando, Florida – EUA

**Keywords:** Embolia Pulmonar, Disfunção Ventricular Direita, Prognóstico, Hemodinâmica, Choque Cardiogênico, Reperfusão, Trombectomia, Modelos Logísticos

## Abstract

**Fundamento:**

A estratificação de risco na embolia pulmonar (EP) aguda baseia-se principalmente nos achados obtidos na admissão hospitalar. No entanto, permanece incerto se a avaliação seriada da disfunção do ventrículo direito (VD) e das alterações hemodinâmicas fornece valor prognóstico incremental além da estratificação de risco inicial.

**Objetivos:**

Avaliar variáveis clínicas e de imagem longitudinais associadas a desfechos hospitalares adversos em pacientes com EP aguda de risco intermediário-alto e alto.

**Métodos:**

Este registro prospectivo incluiu pacientes com EP aguda de risco intermediário-alto e alto. O desfecho primário composto compreendeu mortalidade hospitalar, ventilação mecânica invasiva, choque persistente ou hipotensão e oxigenação por membrana extracorpórea. As variáveis longitudinais foram avaliadas entre 24 e 72 horas após a admissão. Todos os testes estatísticos foram bicaudais, e um valor de p < 0,05 foi considerado estatisticamente significativo. Foram construídos modelos de regressão logística multivariável, e o desempenho discriminativo foi avaliado por meio da área sob a curva característica de operação do receptor (AUC-ROC). Foi realizada uma análise pré-especificada de dados ausentes para avaliar a disponibilidade da tomografia computadorizada (TC) de seguimento.

**Resultados:**

O desfecho primário composto ocorreu em 21,2% dos 151 pacientes, com taxa de mortalidade hospitalar de 9,3%. A disfunção persistente do VD na TC de seguimento (χ^2^ = 15,48; p < 0,001) e a pressão arterial sistólica (PAS) no seguimento (p < 0,001) foram os preditores individuais mais robustos de desfechos adversos. No modelo multivariável longitudinal (n = 60), a disfunção persistente do VD na TC de seguimento (OR 9,70; IC95%, 1,96-47,89; p = 0,005) e a PAS no seguimento (OR 0,90; IC95%, 0,83-0,96; p = 0,003) permaneceram como preditores independentes. O modelo apresentou excelente capacidade discriminativa (AUC-ROC = 0,874) e calibração adequada. A análise de dados ausentes mostrou que os pacientes sem TC de seguimento apresentavam maior gravidade clínica na avaliação inicial, em consonância com um mecanismo de dados ausentes não ao acaso.

**Conclusões:**

A reavaliação de marcadores de risco estabelecidos durante a hospitalização pode aprimorar a identificação de pacientes que permanecem em alto risco apesar da classificação inicial de risco e do tratamento instituído. A disfunção persistente do VD e valores mais baixos de PAS no seguimento estiveram independentemente associados a desfechos hospitalares adversos, o que reforça o uso da estratificação dinâmica de risco na EP aguda.

## Graphical abstract

**Figura Central:**
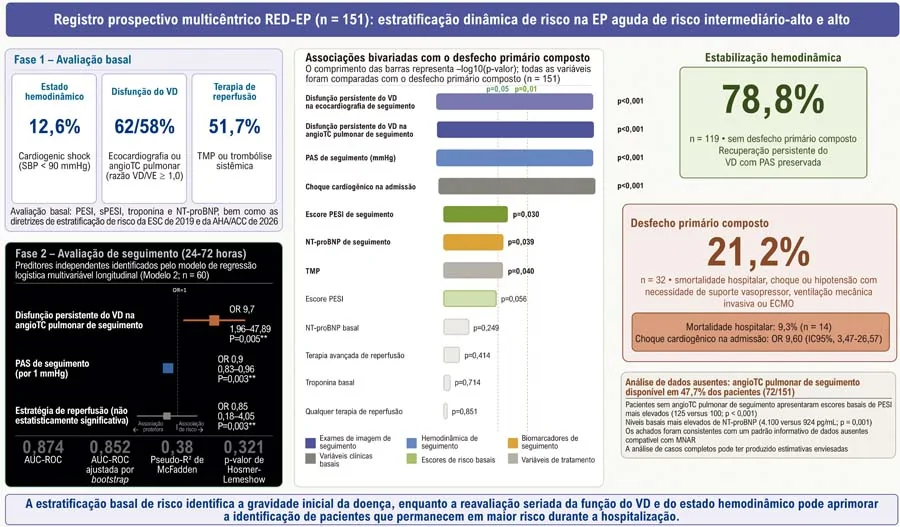
: Disfunção Persistente do Ventrículo Direito e Evolução Hemodinâmica Predizem Deterioração Hospitalar na Embolia Pulmonar de Risco Intermediário-Alto e Alto

A disfunção persistente do VD na angioTC pulmonar de seguimento e valores mais baixos de PAS de seguimento estiveram independentemente associados a desfechos hospitalares adversos. A reavaliação seriada da função do VD e do estado hemodinâmico durante a hospitalização pode aprimorar a estratificação de risco além da avaliação basal inicial em pacientes com EP aguda. AHA/ACC: American Heart Association/American College of Cardiology; angioTC: angiotomografia computadorizada; AUC-ROC: área sob a curva característica de operação do receptor; ECMO: oxigenação por membrana extracorpórea; EP: embolia pulmonar; ESC: European Society of Cardiology; IC95%: intervalo de confiança de 95%; MNAR: dados ausentes não ao acaso; NT-proBNP: fragmento N-terminal do pró-peptídeo natriurético tipo B; OR: odds ratio; PAS: pressão arterial sistólica; PESI: Pulmonary Embolism Severity Index; PMT: trombectomia mecânica percutânea; sPESI: Pulmonary Embolism Severity Index simplificado; VD: ventrículo direito; VE: ventrículo esquerdo.

## Introdução

A embolia pulmonar (EP) aguda permanece como uma das principais causas de morbidade e mortalidade cardiovascular, com incidência global estimada superior a 1-2 casos por 1.000 pessoas-ano.^1,2^ Os modelos contemporâneos de estratificação de risco, incluindo aqueles recomendados pela European Society of Cardiology (ESC) e pela American Heart Association/American College of Cardiology (AHA/ACC), classificam os pacientes de acordo com o estado hemodinâmico, a disfunção do ventrículo direito (VD) detectada por ecocardiografia ou angiotomografia computadorizada (angioTC) pulmonar e biomarcadores cardíacos, incluindo troponina e peptídeos natriuréticos do tipo B (BNP).^1^

O Pulmonary Embolism Severity Index (PESI) e sua versão simplificada (sPESI), ambos desenvolvidos e validados prospectivamente em grandes coortes, fornecem estimativas confiáveis do risco de mortalidade em 30 dias com base exclusivamente em variáveis clínicas.^3,4^ No entanto, uma proporção clinicamente relevante de pacientes classificados como portadores de EP de risco intermediário-alto ou alto apresenta deterioração clínica durante a hospitalização que não é totalmente prevista pela avaliação inicial. O estudo PEITHO evidenciou a heterogeneidade prognóstica existente na população de risco intermediário, demonstrando que a combinação de disfunção do VD e níveis elevados de troponina não identifica de forma consistente os pacientes que posteriormente desenvolverão instabilidade hemodinâmica.^5^

A disfunção do VD, avaliada por ecocardiografia ou angioTC pulmonar, é um preditor independente de desfechos adversos. Algumas metanálises demonstraram que a disfunção do VD identificada por ecocardiografia está associada a um aumento de 2,29 vezes na mortalidade em curto prazo, enquanto uma razão VD/ventrículo esquerdo (VE) ≥ 1,0 na angioTC pulmonar está associada a um aumento aproximado de 2,5 vezes no risco de mortalidade.^6,7^ As troponinas cardíacas e o fragmento N-terminal proBNP (NT-proBNP) aprimoram adicionalmente a estratificação de risco em pacientes normotensos.^8-11^

O conceito de estratificação dinâmica de risco, baseado na avaliação seriada de parâmetros hemodinâmicos, da função do VD e de biomarcadores cardíacos durante a hospitalização, tem recebido crescente atenção. A persistência da disfunção do VD em exames de imagem de seguimento pode refletir obstrução vascular pulmonar não resolvida, recuperação hemodinâmica tardia ou comprometimento circulatório progressivo.^1,2^ Paralelamente, os avanços nas terapias de reperfusão por cateter, incluindo a trombectomia mecânica percutânea (TMP) com dispositivos como o FlowTriever, avaliado nos estudos FLARE e FLAME, e o sistema Penumbra, ampliaram as opções terapêuticas para pacientes com EP de alto risco. Entretanto, em estudos observacionais, a relação entre a estratégia de reperfusão e os desfechos clínicos permanece de difícil interpretação devido ao viés de indicação.^5,12-14^

O registro RED-EP é uma coorte prospectiva multicêntrica estabelecida para caracterizar a apresentação clínica, as estratégias terapêuticas e os desfechos de pacientes com EP de risco intermediário-alto e alto em cenários reais da América Latina. A presente análise investigou se as alterações longitudinais dos parâmetros hemodinâmicos e da função do VD fornecem informações prognósticas adicionais àquelas obtidas pela estratificação de risco baseada na admissão, bem como se a estratégia de reperfusão está independentemente associada aos desfechos hospitalares após ajuste para a gravidade da doença.

## Métodos

### Delineamento do estudo e população

Este estudo de coorte prospectivo, multicêntrico e observacional incluiu pacientes consecutivos com EP aguda confirmada por angioTC pulmonar e classificada como de risco intermediário-alto ou alto em centros participantes das Américas durante um período de 12 meses, de abril de 2025 a abril de 2026. Os dados foram coletados prospectivamente por meio da plataforma mawe. A EP de alto risco foi definida de acordo com as diretrizes da ESC de 2019 como instabilidade hemodinâmica, incluindo choque cardiogênico ou hipotensão sustentada (pressão arterial sistólica [PAS] < 90 mmHg ou redução ≥ 40 mmHg com duração superior a 15 minutos que não pudesse ser explicada por arritmia de início recente, hipovolemia ou sepse).^1^ A EP de risco intermediário-alto foi definida pela presença concomitante de disfunção do VD em exames de imagem (angioTC pulmonar e/ou ecocardiografia) e elevação de biomarcadores cardíacos (troponina e/ou BNP ou NT-proBNP) em pacientes hemodinamicamente estáveis.^1^

### Objetivos do estudo

O objetivo primário foi avaliar variáveis longitudinais associadas a desfechos hospitalares adversos em pacientes com EP aguda de risco intermediário-alto e alto, independentemente da realização de terapia de reperfusão. As estratégias de reperfusão foram selecionadas de acordo com os protocolos institucionais e a disponibilidade de recursos locais nos centros participantes.

Os objetivos secundários foram: i) comparar variáveis da admissão e variáveis longitudinais como preditoras de desfechos adversos; ii) avaliar a associação entre estratégias específicas de reperfusão e desfechos clínicos; e iii) realizar uma análise exploratória dos desfechos de acordo com o subtipo de dispositivo de TMP.

### Definição das variáveis

As variáveis da admissão incluíram idade, sexo, peso corporal, altura, comorbidades, frequência cardíaca, PAS, SpO_2_, PESI,^3^ sPESI,^4^ escore Registro Informatizado de Enfermedad TromboEmbólica, troponina ultrassensível, NT-proBNP, BNP, dímero D, disfunção basal do VD na angioTC pulmonar (definida como razão VD/VE ≥ 1,0 na reconstrução axial de quatro câmaras),^6^ disfunção basal do VD na ecocardiografia e presença de choque cardiogênico.

As variáveis longitudinais foram avaliadas entre 24 e 72 horas após a admissão hospitalar e incluíram frequência cardíaca, PAS, SpO_2_, disfunção do VD na angioTC pulmonar e na ecocardiografia de seguimento, além de novas dosagens de troponina e NT-proBNP. A angioTC pulmonar e a ecocardiografia de seguimento foram realizadas a critério dos médicos assistentes e não eram obrigatórias pelo protocolo do estudo. Consequentemente, os exames de imagem de seguimento foram analisados como uma variável categórica (disponíveis ou indisponíveis entre 24 e 72 horas após a admissão), em vez de serem analisados de acordo com o momento exato de sua realização. A mediana do tempo até a avaliação de seguimento dentro desse intervalo não foi registrada de forma sistemática entre os centros participantes.

As variáveis de tratamento incluíram realização de qualquer terapia de reperfusão, terapia avançada de reperfusão (trombólise sistêmica, trombólise dirigida por cateter ou TMP), tipo de dispositivo de trombectomia (FlowTriever, Penumbra, cateter *pigtail* ou outro) e uso de oxigenação por membrana extracorpórea (ECMO).

### Desfecho primário composto

O desfecho primário composto compreendeu: i) mortalidade hospitalar por todas as causas; ii) choque persistente ou hipotensão com necessidade de suporte vasopressor; iii) necessidade de ventilação mecânica invasiva; e iv) ECMO. Esses componentes representam um continuum clínico de deterioração hemodinâmica em pacientes com EP de alto risco. O choque persistente ou a hipotensão com necessidade de suporte vasopressor refletem insuficiência circulatória persistente e constituem um marcador substituto bem estabelecido de mortalidade nessa população, particularmente entre pacientes submetidos à reperfusão devido à gravidade da apresentação clínica. Os componentes individuais do desfecho composto também foram analisados como desfechos secundários.^15-17^

O desfecho composto foi escolhido para captar manifestações clinicamente relevantes de deterioração hemodinâmica durante a hospitalização. Embora os componentes individuais apresentem diferentes graus de gravidade clínica, todos refletem a incapacidade de alcançar estabilização hemodinâmica após o diagnóstico e o tratamento da EP aguda e representam estágios progressivos de um processo fisiopatológico compartilhado, caracterizado por disfunção persistente do VD, comprometimento circulatório e hipoperfusão de órgãos-alvo.

### Análise estatística

#### Avaliação da normalidade e estatística descritiva

A distribuição das variáveis contínuas foi avaliada por inspeção visual de histogramas e gráficos quantil-quantil (Q-Q), sendo confirmada pelo teste de Shapiro-Wilk. Todas as principais variáveis contínuas apresentaram distribuição não normal, com assimetria à direita. Assim, as variáveis contínuas são apresentadas como mediana e intervalo interquartil (IIQ, percentis 25º-75º). As variáveis categóricas são apresentadas como frequências e porcentagens. Os dados ausentes foram informados para cada variável, e nenhuma imputação foi realizada nas análises primárias.

#### Análise bivariada

As associações entre variáveis categóricas e o desfecho primário composto foram avaliadas por meio do teste do qui-quadrado de independência de Pearson. O teste exato de Fisher foi aplicado quando a frequência esperada em qualquer célula foi < 5. O tamanho do efeito foi quantificado pelo V de Cramér.

As variáveis contínuas foram comparadas por meio do teste *U* de Mann-Whitney (teste da soma dos postos de Wilcoxon), em razão de suas distribuições não normais. Os resultados são apresentados como mediana e IIQ (percentis 25º-75º). O tamanho do efeito para as variáveis contínuas foi calculado por meio da correlação bisserial por postos, definida como $r=1-(2U)/\left(n_{1}\times n_{2}\right)$ . Todos os testes estatísticos foram bicaudais, e um valor de p < 0,05 foi considerado estatisticamente significativo.

#### Regressão logística multivariável

Foram ajustados dois modelos de regressão logística multivariável utilizando estimação por máxima verossimilhança. A função de ligação logit foi utilizada para modelar o logaritmo das chances do desfecho primário de acordo com a equação: $\mathrm{logit}(P)=\beta_{0}+\beta_{1}X_{1}+\beta_{2}X_{2}+\ldots +\beta_{k}X_{k}$ . As *odds ratios* (OR) e seus respectivos intervalos de confiança de 95% (IC95%) foram obtidos pela exponenciação dos coeficientes de regressão, e a significância estatística foi avaliada pelo teste de Wald.^18^

O Modelo 1 (modelo de admissão) incluiu choque cardiogênico na admissão, disfunção do VD na ecocardiografia de seguimento, PAS de seguimento e estratégia de reperfusão (n = 106 casos completos). O Modelo 2 (modelo longitudinal) incluiu disfunção persistente do VD na angioTC pulmonar de seguimento, PAS de seguimento e estratégia de reperfusão (n = 60 casos completos). O choque cardiogênico na admissão foi intencionalmente excluído do Modelo 2 para evitar potencial viés de colisor decorrente da inclusão do choque hospitalar como componente do desfecho primário composto.

As variáveis consideradas clinicamente relevantes e/ou associadas ao desfecho primário nas análises bivariadas (p < 0,10) foram consideradas para inclusão nos modelos multivariáveis. A seleção final dos modelos também levou em consideração o número limitado de eventos do desfecho, a fim de reduzir o risco de sobreajuste. Para minimizar multicolinearidade e redundância, variáveis que representavam construtos clínicos sobrepostos não foram incluídas simultaneamente no mesmo modelo, como por exemplo PAS e choque cardiogênico, ou múltiplos biomarcadores que refletem lesão miocárdica ou estresse cardíaco.^19-21^

#### Desempenho e calibração dos modelos

O desempenho global dos modelos foi avaliado por meio do teste da razão de verossimilhança, do pseudo-R^2^ de McFadden e do critério de informação de Akaike. Um valor de pseudo-R^2^ de McFadden ≥ 0,20 foi considerado indicativo de excelente ajuste do modelo. A capacidade discriminativa foi avaliada por meio da área sob a curva característica de operação do receptor (AUC-ROC).

A calibração dos modelos foi avaliada pelo teste de qualidade de ajuste de Hosmer-Lemeshow. Para avaliar o potencial de sobreajuste e o otimismo no desempenho aparente dos modelos, foi realizada validação interna por reamostragem *bootstrap* com 1.000 iterações. Foram calculadas estimativas da AUC-ROC ajustadas para otimismo, bem como a inclinação e o intercepto da calibração, para avaliar a estabilidade dos modelos e a confiabilidade esperada das predições de risco em populações independentes.

#### Análise de dados ausentes

A angioTC pulmonar de seguimento estava disponível para 72 dos 151 pacientes (47,7%). Foi realizada uma análise pré-especificada de dados ausentes comparando as características basais entre pacientes com e sem angioTC pulmonar de seguimento, com o objetivo de investigar se o mecanismo de dados ausentes era compatível com dados completamente ausentes ao acaso, dados ausentes ao acaso ou dados ausentes não ao acaso (*missing not at random* [MNAR]).^22,23^ As variáveis contínuas foram comparadas por meio do teste *U* de Mann-Whitney, e as variáveis categóricas pelo teste do qui-quadrado de Pearson.

#### Análise de subgrupos de reperfusão

As características basais e os desfechos clínicos foram comparados descritivamente entre os grupos de estratégia de reperfusão (sem reperfusão, trombólise sistêmica e TMP) para caracterizar o potencial confundimento por indicação.^5,12,13^ Também foi realizada uma comparação exploratória de acordo com o dispositivo de trombectomia utilizado (FlowTriever, Penumbra ou cateter *pigtail*).

#### Software

Todas as análises estatísticas foram realizadas no JASP versão 0.97.0. Os testes não paramétricos, as análises de regressão logística e as análises da curva ROC foram conduzidos no JASP. As análises confirmatórias e a geração das figuras foram realizadas em Python 3.12 utilizando o Matplotlib versão 3.8.0. A geração de código assistida por inteligência artificial foi utilizada durante o processo de programação, e todo o código, as análises e os resultados foram revisados e verificados de forma independente pelos autores.

## Resultados

### Características da coorte

Um total de 151 pacientes foi incluído na análise. O desfecho primário composto ocorreu em 32 pacientes (21,2%), e a taxa de mortalidade hospitalar foi de 9,3% (n = 14). As características basais, as variáveis longitudinais e os dados de tratamento, estratificados de acordo com a ocorrência do desfecho primário composto, são apresentados na Tabela 1 e na Figura 1.

**Tabela 1:** – Características basais, variáveis de seguimento e desfechos clínicos de acordo com o desfecho primário composto

Variável	Geral (n = 151)	Sem evento (n = 119)	Com evento (n = 32)	Valor de p
**Dados demográficos e escores clínicos**				
Idade, mediana (IIQ), anos	67 (57-76)	66 (57-76)	68 (58-75)	0,440
Sexo masculino, n (%)	67 (44,4)	55 (46,2)	12 (37,5)	0,378
Escore PESI, mediana (IIQ)	110 (79-138)	105 (77-136)	124 (89-158)	0,061
Escore sPESI, mediana (IIQ)	2 (1-3)	2 (1-3)	3 (1-3)	0,001
EP de alto risco (choque cardiogênico), n (%)	19 (12,6)	7 (5,9)	12 (37,5)	< 0,001
**Imagem basal e biomarcadores**				
Disfunção do VD na ecocardiografia, n (%)	93 (62,0)	70 (63,1)	23 (71,9)	0,357
Razão VD/VE > 1,0 na angioTC pulmonar, n (%)	87 (57,6)	65 (54,6)	22 (68,8)	0,151
Troponina, mediana (IIQ), ng/L	98 (35-188)	91 (35-188)	112 (40-189)	0,347
NT-proBNP, mediana (IIQ), pg/mL	2.812 (680-5.130)	2.450 (675-4.515)	3.927 (1.063-7.562)	0,105
Dímero D, mediana (IIQ), µg/L	9.800 (4.270-14.023)	9.800 (4.270-14.023)	7.525 (1.834-11.765)	0,126
**Estratégias de tratamento**				
Qualquer terapia de reperfusão, n (%)	78 (51,7)	61 (51,3)	17 (53,1)	0,851
Terapia avançada de reperfusão, n (%)	71 (47,0)	58 (48,7)	13 (40,6)	0,414
TMP, n (%)	46 (36,8)	44/99 (44,4)	2/18 (11,1)	0,040
Trombólise sistêmica, n (%)	24 (15,9)	17 (14,3)	7 (21,9)	0,113
**Variáveis de seguimento (24-72 h)**				
PAS de seguimento, mediana (IIQ), mmHg	—	125 (115-130)	103 (100-127)	< 0,001
Disfunção persistente do VD na angioTC pulmonar de seguim ento, n (%)	22/72 (30,6)	9/50 (18,0)	13/22 (59,1)	< 0,001
Disfunção persistente do VD na ecocardiografia de seguimento, n (%)	39/114 (34,2)	23/75 (30,7)	16/39 (41,0)	0,001
NT-proBNP de seguimento, mediana (IIQ), pg/mL	—	656 (197-1.678)	1.740 (920-7.555)	0,039
**Desfechos clínicos**				
Mortalidade hospitalar, n (%)	14 (9,3)	0 (0)	14 (43,8)	N/A
Choque ou hipotensão com necessidade de vasopressores, n (%)	22 (14,6)	0 (0)	22 (68,8)	N/A
Ventilação mecânica invasiva, n (%)	6 (4,0)	0 (0)	6 (18,8)	N/A
ECMO, n (%)	7 (4,6)	0 (0)	7 (21,9)	N/A
Desfecho primário composto, n (%)	32 (21,2)	0 (0)	32 (100)	N/A

AngioTC: angiotomografia computadorizada; ECMO: oxigenação por membrana extracorpórea; EP: embolia pulmonar; IIQ: intervalo interquartil; N/A: não aplicável; NT-proBNP: fragmento N-terminal do pró-peptídeo natriurético tipo B; PAS: pressão arterial sistólica; PESI: Pulmonary Embolism Severity Index; sPESI: versão simplificada do Pulmonary Embolism Severity Index; TMP: trombectomia mecânica percutânea; VD: ventrículo direito; VE: ventrículo esquerdo. As variáveis contínuas são apresentadas como mediana (IIQ [percentis 25º-75º]), e as variáveis categóricas como n (%). As variáveis contínuas foram comparadas por meio do teste U de Mann-Whitney, enquanto as variáveis categóricas foram comparadas pelo teste do qui-quadrado de Pearson ou pelo teste exato de Fisher, conforme apropriado. Os denominadores das variáveis de seguimento variam de acordo com a disponibilidade dos dados: a angioTC pulmonar de seguimento estava disponível para 72 dos 151 pacientes (47,7%), e a ecocardiografia de seguimento para 114 dos 151 pacientes (75,5%). O denominador para TMP corresponde ao número de pacientes com dados do procedimento disponíveis (n = 125).

**Figura 1:**
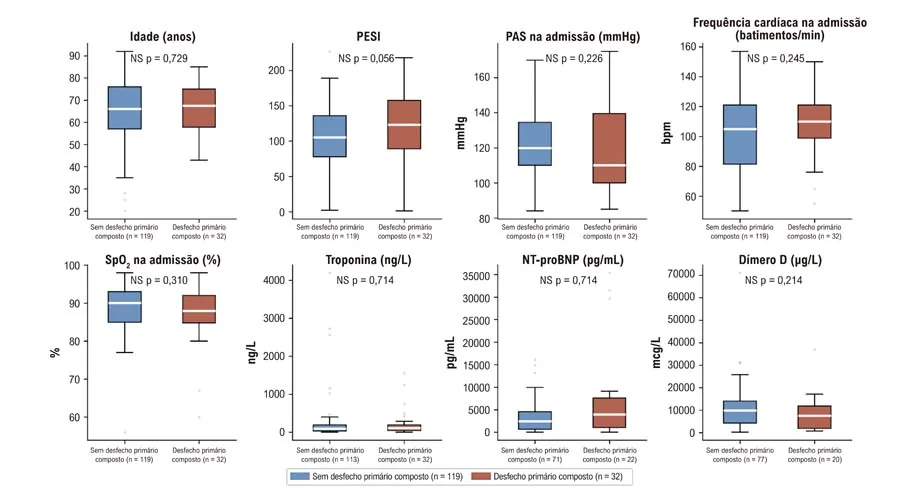
– Características clínicas e de biomarcadores basais de acordo com o desfecho primário composto. Boxplots comparando variáveis clínicas, hemodinâmicas e biomarcadores basais entre pacientes que apresentaram o desfecho primário composto (vermelho) e aqueles que não o apresentaram (azul) (n = 151). As variáveis contínuas foram comparadas por meio do teste U de Mann-Whitney bicaudal. As caixas representam o IIQ, a linha horizontal indica a mediana, os whiskers estendem-se até 1,5 × IIQ e os pontos representam valores atípicos. ***p < 0,001; **p < 0,01; *p < 0,05. IIQ: intervalo interquartil; NS: não significativo; NT-proBNP: fragmento N-terminal do pró-peptídeo natriurético tipo B; PAS: pressão arterial sistólica; PESI: Pulmonary Embolism Severity Index.

### Análise bivariada do desfecho primário composto

A análise bivariada completa é apresentada nas Tabelas 2, 3 e 4, uma análise multivariada de variáveis contínuas é representada na Figura 2. Entre as variáveis de imagem, a disfunção persistente do VD na angioTC pulmonar de seguimento foi o mais forte preditor categórico do desfecho primário composto (χ^2^ = 15,48, graus de liberdade [gl] = 1, p < 0,001; OR 6,95).^6,7^ Entre as variáveis contínuas, a PAS de seguimento apresentou a associação mais forte com desfechos adversos (teste *U* de Mann-Whitney, p < 0,001).

**Tabela 2:** – Características basais de acordo com o status de reperfusão

Variável	Sem reperfusão (n = 73)	Reperfusão (n = 78)	Valor de p
Idade, anos, mediana (IIQ)	69 (57-79)	65 (57-73)	0,574
Peso corporal, kg, mediana (IIQ)	75 (68-83)	84 (75-95)	0,336
Altura, cm, mediana (IIQ)	168 (160-172)	170 (165-176)	0,015^*^
Escore PESI, mediana (IIQ)	100 (70-123)	126 (90-150)	0,040^*^
Escore sPESI, mediana (IIQ)	2 (1-3)	2 (1-3)	0,333
Frequência cardíaca, batimentos/min, mediana (IIQ)	90 (80-110)	118 (96-125)	0,237
PAS, mmHg, mediana (IIQ)	120 (110-135)	120 (109-135)	0,328
SpO_2_, %, mediana (IIQ)	92 (89-95)	86 (84-90)	0,165
Disfunção persistente do VD na angioTC pulmonar de seguimento, n (%)	7/50 (14,0)	13/22 (59,1)	< 0,001^***^
Choque cardiogênico na admissão, n (%)	20/132 (15,1)	12/19 (63,2)	< 0,001^***^
Desfecho primário composto, n (%)	15 (20,5)	17 (21,8)	0,851
Mortalidade hospitalar, n (%)	8 (11,0)	6 (7,7)	0,489

AngioTC: angiotomografia computadorizada; IIQ: intervalo interquartil; PAS: pressão arterial sistólica; PESI: Pulmonary Embolism Severity Index; sPESI: Pulmonary Embolism Severity Index simplificado; VD: ventrículo direito. As variáveis contínuas são apresentadas como mediana (IIQ [percentis 25º-75º]), e as variáveis categóricas como n (%). As variáveis contínuas foram comparadas por meio do teste U de Mann-Whitney bicaudal, e as variáveis categóricas pelo teste do qui-quadrado de Pearson. *p < 0,05; ***p < 0,001. O denominador para a disfunção persistente do VD na angioTC pulmonar de seguimento corresponde ao número de pacientes submetidos à repetição da angioTC pulmonar.

**Tabela 3:** – Análise bivariada das variáveis qualitativas associadas ao desfecho primário composto

Variável	Sem evento (n = 119)	Com evento (n = 32)	Valor de p	OR (IC95%)
Disfunção basal do VD na ecocardiografia, n (%)	70 (63,1)	23 (71,9)	0,357	—
Razão basal VD/VE > 1,0 na angioTC pulmonar, n (%)	65 (54,6)	22 (68,8)	0,151	—
Qualquer terapia de reperfusão, n (%)	58 (48,7)	15 (46,9)	0,851	0,93 (0,43-2,01)
Choque cardiogênico na admissão, n (%)	7 (5,9)	12 (37,5)	< 0,001^***^	9,60 (3,47-26,57)
Disfunção persistente do VD na angioTC pulmonar de seguimento, n (%)^†^	9 (18,0)	13 (59,1)	< 0,001^***^	6,95 (2,39-20,21)
Disfunção persistente do VD na ecocardiografia de seguimento, n (%)^‡^	23 (30,7)	16 (61,5)	< 0,001^***^	3,62 (1,48-8,86)

AngioTC: angiotomografia computadorizada; gl: graus de liberdade; IC95%: intervalo de confiança de 95%; OR: odds ratio; VD: ventrículo direito; VE: ventrículo esquerdo. Os dados são apresentados como n (%). Os valores de p foram calculados por meio do teste do qui-quadrado de Pearson (gl = 1, bicaudal). O teste exato de Fisher foi utilizado quando a frequência esperada em qualquer célula foi < 5. ^†^AngioTC pulmonar de seguimento disponível para 72 pacientes. ^‡^Ecocardiografia de seguimento disponível para 114 pacientes. ***p < 0,001.

**Tabela 4:** – Análise bivariada das variáveis quantitativas associadas ao desfecho primário composto

Variável	Sem evento (n = 119)	Com evento (n = 32)	Valor de p
Altura, cm, mediana (IIQ)	170 (162-176)	165 (162-170)	0,015^*^
PAS de seguimento, mmHg, mediana (IIQ)	124 (115-130)	102 (100-127)	< 0,001^***^
SpO_2_ de seguimento, %, mediana (IIQ)	95 (93-96)	94 (92-95)	0,013^*^
Troponina, ng/L, mediana (IIQ)	91 (35-188)	112 (40-188)	0,014^*^
NT-proBNP de seguimento, pg/mL, mediana (IIQ)	656 (197-1.678)	1.740 (920-7.555)	0,039^*^

IIQ: intervalo interquartil; NT-proBNP: fragmento N-terminal do pró-peptídeo natriurético tipo B; PAS: pressão arterial sistólica. As variáveis contínuas são apresentadas como mediana (IIQ [percentis 25º-75º]). As comparações entre os grupos foram realizadas por meio do teste U de Mann-Whitney bicaudal. *p < 0,05; ***p < 0,001.

**Figura 2:**
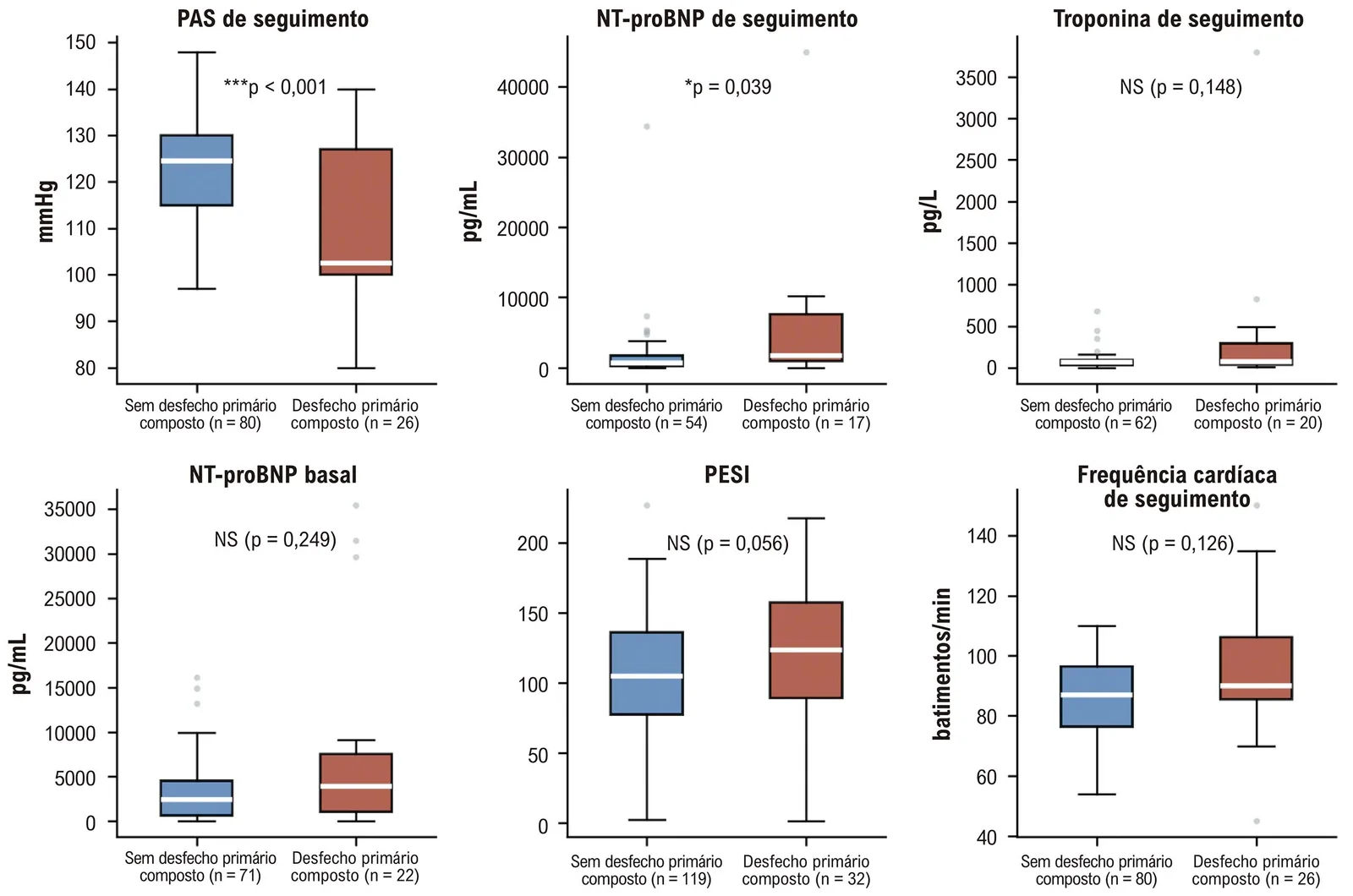
– Distribuição das principais variáveis contínuas de acordo com o desfecho primário composto. Boxplots comparando variáveis contínuas basais e de seguimento (24-72 horas) entre pacientes que apresentaram o desfecho primário composto (vermelho) e aqueles que não o apresentaram (azul). A PAS de seguimento e o NT-proBNP de seguimento estiveram significativamente associados ao desfecho primário composto. As caixas representam o IIQ, a linha horizontal indica a mediana, os whiskers estendem-se até 1,5 × IIQ e os pontos representam valores atípicos. As comparações estatísticas foram realizadas por meio do teste U de Mann-Whitney bicaudal. ***p < 0,001; *p < 0,05. Os tamanhos amostrais são apresentados abaixo de cada boxplot. IIQ: intervalo interquartil; NS: não significativo; NT-proBNP: fragmento N-terminal do pró-peptídeo natriurético tipo B; PAS: pressão arterial sistólica; PESI: Pulmonary Embolism Severity Index.

Nenhuma estratégia de reperfusão esteve significativamente associada ao desfecho primário composto nas análises bivariadas. Os achados de imagem basais e os biomarcadores cardíacos, em geral, não apresentaram associação significativa com desfechos adversos nesta coorte, enquanto diversas variáveis longitudinais demonstraram associações mais fortes e consistentes com o desfecho primário composto.^8-11^

### Regressão logística multivariável

Foram desenvolvidos dois modelos de regressão logística multivariável (Tabela 5 e Figura 3). Em ambos os modelos, a PAS de seguimento permaneceu como preditor independente do desfecho primário composto. No modelo longitudinal (Modelo 2), a disfunção persistente do VD na angioTC pulmonar de seguimento também permaneceu independentemente associada a desfechos hospitalares adversos.

**Tabela 5:** – Análise de regressão logística multivariável dos preditores independentes do desfecho primário composto

Variável	Coeficiente β	EP	Estatística z de Wald	OR (IC95%)	Valor de p
**Modelo 1 – Modelo de admissão**					
n = 106; pseudo-R^2^ de McFadden = 0,301; AUC-ROC = 0,807; χ^2^ da RV = 35,54; p da RV < 0,001; AIC = 92,6					
Intercepto	6,64	2,69	2,47	765,7 (N/A)	0,014
Choque cardiogênico na admissão	2,38	0,75	3,19	10,82 (2,51-46,69)	0,001^**^
Disfunção persistente do VD na ecocardiografia de seguimento	0,73	0,69	1,07	2,08 (0,54-7,98)	0,286
PAS de seguimento (por 1 mmHg)	–0,071	0,023	–3,06	0,93 (0,89-0,97)	0,002^**^
Estratégia de reperfusão	–0,80	0,66	–1,22	0,45 (0,12-1,63)	0,224
**Modelo 2 – Modelo longitudinal**					
n = 60; pseudo-R^2^ de McFadden = 0,381; AUC-ROC = 0,874; χ^2^ de Hosmer-Lemeshow = 7,00 (gl = 6; p = 0,321); χ^2^ da RV = 27,96; p da RV < 0,001; AIC = 53,3					
Intercepto	10,61	4,13	2,57	N/A	0,010
Disfunção persistente do VD na angioTC pulmonar de seguimento	2,27	0,81	2,79	9,70 (1,96-47,89)	0,005^**^
PAS de seguimento (por 1 mmHg)	–0,108	0,037	–2,95	0,90 (0,83-0,96)	0,003^**^
Estratégia de reperfusão	–0,17	0,80	–0,21	0,85 (0,18-4,05)	0,836

AIC: critério de informação de Akaike; angioTC: angiotomografia computadorizada; AUC-ROC: área sob a curva característica de operação do receptor; EP: erro-padrão; gl: graus de liberdade; IC95%: intervalo de confiança de 95%; N/A: não aplicável; OR: odds ratio; PAS: pressão arterial sistólica; RV: razão de verossimilhança; VD: ventrículo direito. Os modelos de regressão logística multivariável foram ajustados utilizando estimação por máxima verossimilhança. As OR foram obtidas pela exponenciação dos coeficientes de regressão (OR = exp[β]), e os IC95% foram calculados como exp(β ± 1,96 × EP). A significância estatística foi avaliada pelo teste de Wald (z = β/EP). Valores de pseudo-R2 de McFadden ≥ 0,20 foram considerados indicativos de excelente ajuste do modelo. A calibração foi avaliada pelo teste de qualidade de ajuste de Hosmer-Lemeshow com oito grupos (gl = 6); p > 0,05 indica calibração adequada. A capacidade discriminativa foi avaliada pela AUC-ROC com base nas predições aparentes (na própria amostra). **p < 0,01.

**Figura 3:**
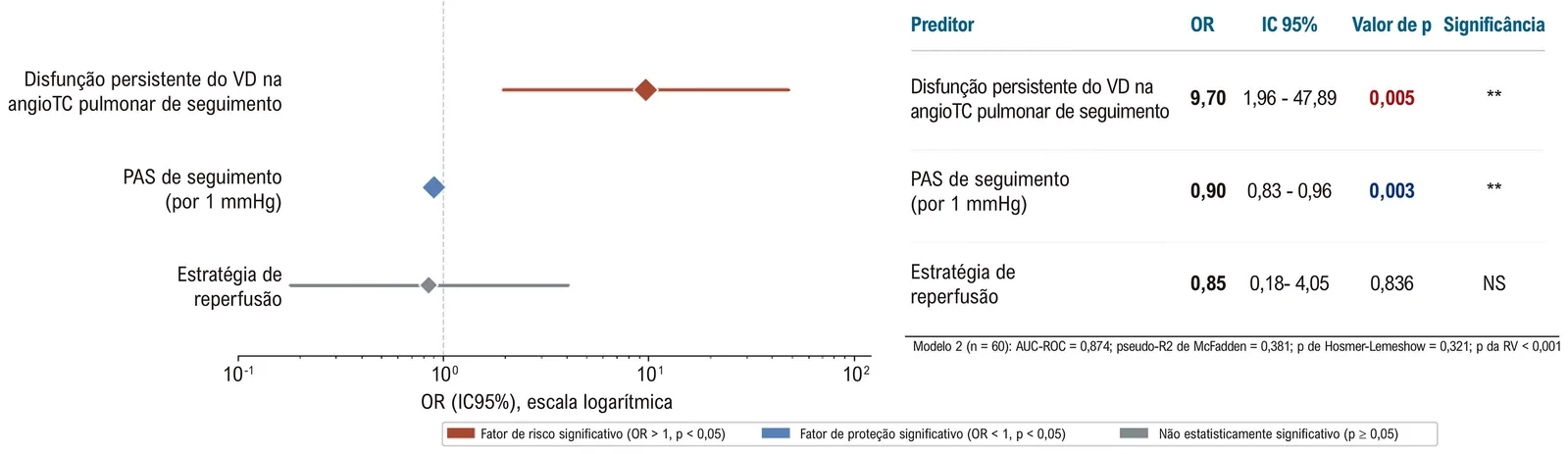
– Forest plot do modelo de regressão logística multivariável longitudinal (Modelo 2). Forest plot apresentando as OR ajustadas e os IC95% dos preditores independentes do desfecho primário composto identificados no modelo de regressão logística multivariável longitudinal. O modelo foi ajustado utilizando estimação por máxima verossimilhança (n = 60 casos completos). As OR foram calculadas como exp(β), e os IC95% como exp(β ± 1,96 × EP). A linha vertical de referência indica OR = 1,0. Losangos vermelhos representam fatores de risco estatisticamente significativos (OR > 1; p < 0,05), losangos azuis representam fatores de proteção estatisticamente significativos (OR < 1; p < 0,05) e losangos cinza representam associações não significativas. Desempenho do modelo: AUC-ROC = 0,874; pseudo-R2 de McFadden = 0,381; p de Hosmer-Lemeshow = 0,321; p da RV < 0,001. AngioTC: angiotomografia computadorizada; AUC-ROC: área sob a curva característica de operação do receptor; EP: erro-padrão; IC95%: intervalo de confiança de 95%; NS: não significativo; OR: odds ratio; PAS: pressão arterial sistólica; RV: razão de verossimilhança; VD: ventrículo direito.

De modo geral, o modelo longitudinal demonstrou capacidade discriminativa e calibração superiores em comparação com o modelo de admissão. A AUC-ROC aparente foi de 0,874, enquanto a AUC ajustada para otimismo obtida por validação *bootstrap* foi de 0,852 (Figura 4). A calibração permaneceu satisfatória após a validação interna, com inclinação de calibração ajustada de 0,86 e intercepto de calibração ajustado de 0,04, indicando boa concordância entre os riscos preditos e observados e evidência mínima de viés sistemático de predição.

**Figura 4:**
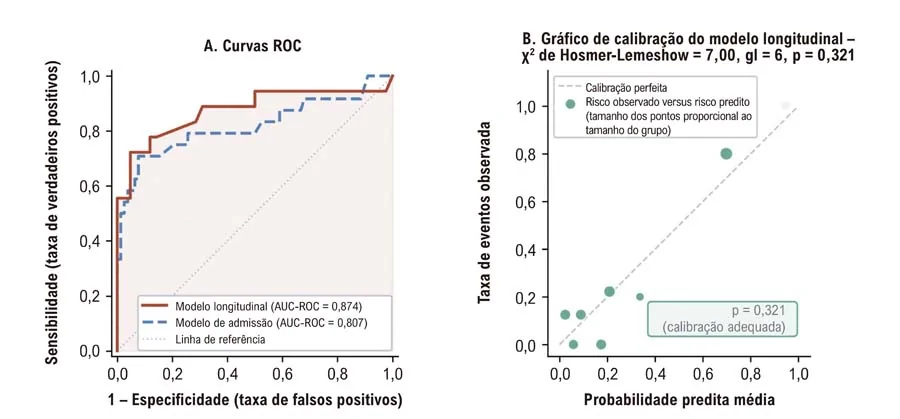
– Discriminação e calibração do modelo preditivo longitudinal. Painel A: Curvas ROC comparando o modelo longitudinal (Modelo 2; AUC-ROC = 0,874, linha vermelha contínua) com o modelo de admissão (Modelo 1; AUC-ROC = 0,807, linha azul tracejada). Painel B: Gráfico de calibração do modelo longitudinal. Cada ponto representa um decil de risco, com tamanho proporcional ao número de pacientes no respectivo grupo. O teste de qualidade de ajuste de Hosmer-Lemeshow demonstrou calibração adequada (χ2 = 7,00; gl = 6; p = 0,321). A AUC-ROC ajustada por bootstrap foi de 0,852, sustentando a robustez da capacidade discriminativa do modelo. AUC-ROC: área sob a curva característica de operação do receptor; gl: graus de liberdade.

### Análise de dados ausentes da disponibilidade da angiotomografia computadorizada pulmonar de seguimento

A angioTC pulmonar de seguimento estava disponível para 72 dos 151 pacientes (47,7%). Em comparação com os pacientes submetidos à repetição do exame de imagem, aqueles sem angioTC pulmonar de seguimento apresentaram, em geral, maior gravidade da doença na avaliação basal (Tabela 6 e Figura 5), sugerindo um mecanismo informativo de dados ausentes compatível com MNAR.^22,23^ Esses achados indicam que a decisão de realizar o exame de imagem de seguimento foi influenciada pela evolução clínica do paciente, e não ocorreu de forma aleatória. As taxas de choque cardiogênico na admissão, do desfecho primário composto e de terapia de reperfusão não diferiram significativamente entre os grupos.

**Tabela 6:** – Análise de dados ausentes de acordo com a disponibilidade da angioTC pulmonar de seguimento

Variável	AngioTC pulmonar de seguimento disponível (n = 72)	AngioTC pulmonar de seguimento indisponível (n = 79)	Valor de p
**Variáveis contínuas**			
Idade, anos, mediana (IIQ)	68 (56-76)	66 (58-72)	0,912
Escore PESI, mediana (IIQ)	100 (53-124)	125 (100-150)	< 0,001^***^
Escore sPESI, mediana (IIQ)	2 (1-3)	2 (1-3)	0,010^*^
PAS na admissão, mmHg, mediana (IIQ)	118 (105-130)	126 (110-138)	0,022^*^
**Frequência cardíaca na admissão, batimentos/min, mediana (IIQ)**	92 (80-115)	110 (100-122)	0,001^***^
**SpO_**2**_ na admissão, %, mediana (IIQ)**	90 (86-94)	87 (85-92)	0,018^*^
**Troponina, ng/L, mediana (IIQ)**	52 (13-174)	134 (73-188)	0,002^**^
**NT-proBNP, pg/mL, mediana (IIQ)**	924 (98-3.814)	4.100 (1.895-5.800)	0,001^***^
**Variáveis categóricas**			
Choque cardiogênico na admissão, n (%)	12 (16,7)	7 (8,9)	0,231
Desfecho primário composto, n (%)	20 (27,8)	12 (15,2)	0,091
Qualquer terapia de reperfusão, n (%)	34 (47,2)	44 (55,7)	0,380
Disfunção basal do VD na ecocardiografia, n (%)	39 (54,2)	55 (69,2)	0,084

AngioTC: angiotomografia computadorizada; IIQ: intervalo interquartil; NT-proBNP: fragmento N-terminal do pró-peptídeo natriurético tipo B; PAS: pressão arterial sistólica; PESI: Pulmonary Embolism Severity Index; sPESI: Pulmonary Embolism Severity Index simplificado; VD: ventrículo direito. As variáveis contínuas são apresentadas como mediana (IIQ [percentis 25º-75º]), e as variáveis categóricas como n (%). As variáveis contínuas foram comparadas por meio do teste U de Mann-Whitney bicaudal, enquanto as variáveis categóricas foram comparadas pelo teste do qui-quadrado de Pearson. ***p < 0,001; **p < 0,01; *p < 0,05.

**Figura 5:**
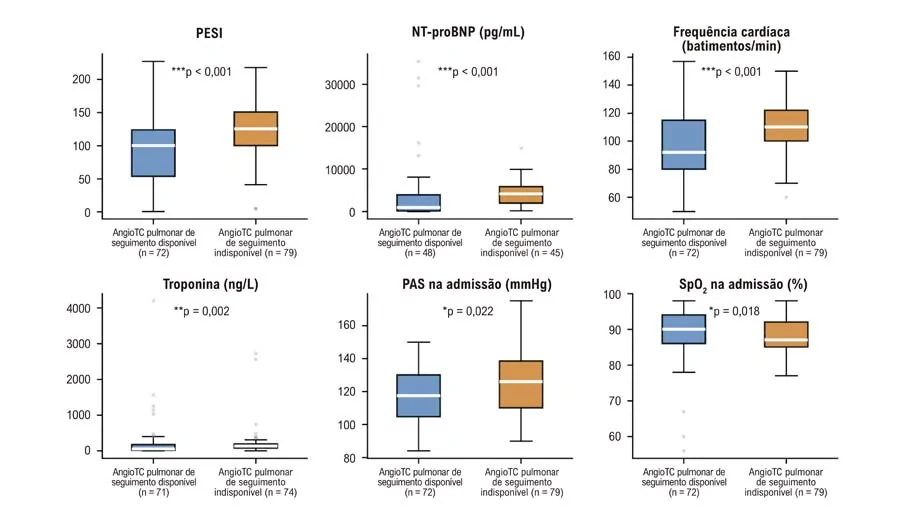
– Características basais de acordo com a disponibilidade da angioTC pulmonar de seguimento. Boxplots comparando variáveis clínicas e biomarcadores basais entre pacientes com angioTC pulmonar de seguimento disponível (azul, n = 72) e aqueles sem angioTC pulmonar de seguimento (laranja, n = 79). Os pacientes sem angioTC pulmonar de seguimento apresentaram escores basais de PESI, frequência cardíaca, troponina e níveis de NT-proBNP mais elevados, compatíveis com um padrão informativo de dados ausentes consistente com MNAR. As caixas representam o IIQ, a linha horizontal indica a mediana, os whiskers estendem-se até 1,5 × IIQ e os pontos representam valores atípicos. As comparações estatísticas foram realizadas por meio do teste U de Mann-Whitney bicaudal. ***p < 0,001; **p < 0,01; *p < 0,05. AngioTC: angiotomografia computadorizada; IIQ: intervalo interquartil; MNAR: dados ausentes não ao acaso; NT-proBNP: fragmento N-terminal do pró-peptídeo natriurético tipo B; PAS: pressão arterial sistólica; PESI: Pulmonary Embolism Severity Index.

O padrão observado de MNAR é provavelmente explicado por dois mecanismos concorrentes. Primeiro, alguns pacientes apresentaram deterioração hemodinâmica precoce e encontravam-se clinicamente instáveis demais para serem submetidos à repetição do exame de imagem. Segundo, pacientes com rápida melhora clínica podem não ter realizado angioTC pulmonar de seguimento porque exames adicionais foram considerados desnecessários pelos médicos assistentes. Em conjunto, esses mecanismos podem ter enriquecido a amostra analisada com pacientes de gravidade clínica intermediária. Consequentemente, a direção e a magnitude do potencial viés nas estimativas derivadas do Modelo 2 não podem ser determinadas sem a aplicação de métodos formais para tratamento de dados ausentes, como imputação múltipla.^22,23^ Esses achados reforçam a necessidade de estudos prospectivos multicêntricos que incorporem angioTC pulmonar de seguimento prevista em protocolo em momentos predefinidos, independentemente da evolução clínica, para definir com maior precisão o valor prognóstico da disfunção persistente do VD e reduzir o potencial viés de seleção relacionado à repetição dos exames de imagem.

### Mortalidade hospitalar

A taxa de mortalidade hospitalar foi de 9,3% (n = 14). Na análise bivariada, a mortalidade esteve significativamente associada ao choque cardiogênico na admissão (χ^2^ = 7,51; p = 0,006), ao choque persistente ou hipotensão com necessidade de suporte vasopressor durante a hospitalização (χ^2^ = 5,54; p = 0,019), à disfunção do VD na ecocardiografia (χ^2^ = 4,57; p = 0,032) e à necessidade de ventilação mecânica invasiva (χ^2^ = 4,30; p = 0,038). Em contrapartida, nem a razão basal VD/VE na angioTC pulmonar (p = 0,970) nem os níveis basais de troponina (p = 0,851) apresentaram associação significativa com a mortalidade hospitalar, reforçando o maior valor prognóstico das variáveis longitudinais em comparação com as medidas obtidas na admissão.^6,8^

### Estratégias de reperfusão

Entre os 78 pacientes (51,7%) submetidos à terapia de reperfusão, 24 (15,9%) receberam trombólise sistêmica e 46 foram submetidos à TMP, predominantemente com o dispositivo FlowTriever (n = 36). Nem a terapia de reperfusão em geral (p = 0,851) nem a terapia avançada de reperfusão (p = 0,414) estiveram independentemente associadas ao desfecho primário composto.^5,12,13^

Conforme apresentado na Tabela 7, a distribuição das características basais entre os grupos de tratamento reflete importante confundimento por indicação. Os pacientes tratados com trombólise sistêmica apresentaram a maior prevalência de choque cardiogênico na admissão (37,5%), enquanto aqueles submetidos à trombectomia mecânica apresentaram escores de PESI e concentrações de NT-proBNP mais elevados, porém menor prevalência de choque cardiogênico (13,0%), compatível com um fenótipo clínico de alto risco, mas hemodinamicamente estável. A incidência numericamente menor do desfecho primário composto no grupo submetido à trombectomia (13,0% versus 20,5% no grupo sem reperfusão) deve, portanto, ser interpretada com cautela, uma vez que provavelmente é atribuível à seleção do tratamento com base nas características clínicas basais, e não a um verdadeiro efeito terapêutico.

**Tabela 7:** – Características basais e desfechos clínicos de acordo com a estratégia de reperfusão

Variável	Sem reperfusão (n = 73)	Trombólise sistêmica (n = 24)	TMP (n = 46)
Choque cardiogênico na admissão, n (%)	3 (4,1)	9 (37,5)	6 (13,0)
Escore PESI, mediana (IIQ)	100 (70-123)	117 (105-148)	137 (116-156)
NT-proBNP, pg/mL, mediana (IIQ)	820 (86-5.805)	3.128 (1.554-6.852)	3.828 (2.201-4.912)
Desfecho primário composto, n (%)	15/73 (20,5)	8/24 (33,3)	6/46 (13,0)
Mortalidade hospitalar, n (%)	8/73 (11,0)	3/24 (12,5)	2/46 (4,3)

IIQ: intervalo interquartil; NT-proBNP: fragmento N-terminal do pró-peptídeo natriurético tipo B; PESI: Pulmonary Embolism Severity Index; TMP: trombectomia mecânica percutânea. Os dados são apresentados de forma descritiva. Não foram realizadas comparações estatísticas formais devido ao pequeno tamanho das amostras e à presença de importante confundimento por indicação. Os grupos de reperfusão não foram mutuamente exclusivos para todos os pacientes.

## Discussão

### Principais achados

Os presentes achados sugerem que marcadores de risco já incorporados aos modelos contemporâneos de estratificação de risco da ESC e da AHA/ACC mantêm substancial valor prognóstico quando reavaliados durante a hospitalização. A avaliação seriada da recuperação do VD e da estabilização hemodinâmica parece fornecer informações prognósticas incrementais em relação às disponíveis na admissão. A disfunção persistente do VD na angioTC pulmonar de seguimento (OR 9,70; IC95%, 1,96-47,89; p = 0,005) e a PAS de seguimento (OR 0,90 por mmHg; IC95%, 0,83-0,96; p = 0,003) emergiram como os principais preditores independentes de desfechos hospitalares adversos, enquanto biomarcadores da admissão, escores de risco estabelecidos e estratégias de reperfusão não estiveram independentemente associados ao desfecho primário composto. Esses achados ampliam o modelo prognóstico proposto pelas diretrizes da ESC de 2019 e pela diretriz da AHA/ACC de 2026 ao destacar a importância da avaliação dinâmica do risco durante a hospitalização.^1,14^ Conforme ilustrado na Figura 6 e Figura Central, pacientes com disfunção persistente do VD acompanhada de evolução hemodinâmica desfavorável apresentaram taxas substancialmente mais elevadas de deterioração clínica hospitalar.

**Figura 6:**
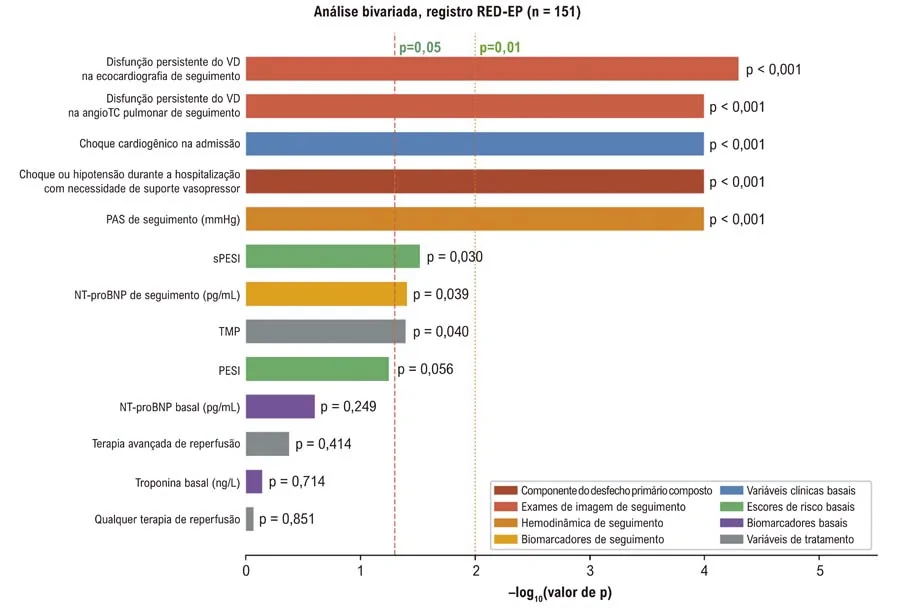
– Significância estatística das variáveis associadas ao desfecho primário composto. O comprimento das barras representa os valores de –log10(valor de p) derivados das análises bivariadas, utilizando o teste do qui-quadrado de Pearson para variáveis categóricas e o teste U de Mann-Whitney bicaudal para variáveis contínuas. A linha vermelha tracejada indica o limiar de significância estatística (p = 0,05), e a linha laranja pontilhada indica p = 0,01. As cores identificam as categorias das variáveis: exames de imagem de seguimento (vermelho), hemodinâmica de seguimento (laranja), biomarcadores de seguimento (dourado), variáveis clínicas basais (azul), escores de risco basais (verde), biomarcadores basais (roxo), componentes do desfecho primário composto (vermelho-escuro) e variáveis de tratamento (cinza). Barras mais longas indicam evidências estatísticas mais fortes de associação com o desfecho primário composto. AngioTC: angiotomografia computadorizada; NT-proBNP: fragmento N-terminal do pró-peptídeo natriurético tipo B; PESI: Pulmonary Embolism Severity Index; PAS: pressão arterial sistólica; PMT: trombectomia mecânica percutânea; sPESI: versão simplificada do Pulmonary Embolism Severity Index; VD: ventrículo direito.

### Justificativa para o desfecho composto e significado clínico do choque/hipotensão

O desfecho composto, compreendendo mortalidade por todas as causas, ventilação mecânica invasiva, ECMO e choque ou hipotensão com necessidade de suporte vasopressor, foi pré-especificado porque esses eventos representam um continuum clínico de falência do VD e descompensação circulatória progressiva.^15-17^ No contexto específico da EP de risco intermediário-alto e alto, em que os pacientes foram considerados candidatos à reperfusão em razão da gravidade do comprometimento hemodinâmico, o choque persistente ou recorrente com necessidade de suporte vasopressor não deve ser considerado um desfecho de menor importância. Ao contrário, ele reflete obstrução vascular pulmonar persistente, falha da recuperação do VD apesar do tratamento e constitui um marcador substituto estabelecido de mortalidade em curto prazo.^1,5^ Esse modelo conceitual é consistente com o estudo PEITHO, no qual a descompensação hemodinâmica foi o principal componente contribuinte para o desfecho composto, e com a diretriz da AHA/ACC sobre EP de 2026, que identifica a incapacidade de manter a estabilidade hemodinâmica como uma característica definidora de falha terapêutica na EP de alto risco.^5,14^

Embora os componentes individuais do desfecho composto apresentem diferentes graus de gravidade clínica, todos indicam falha em alcançar estabilização cardiovascular após o diagnóstico e o tratamento da EP aguda. Em nossa coorte, o choque persistente com necessidade de suporte vasopressor foi o componente mais frequente, enquanto ECMO e ventilação mecânica invasiva ocorreram com menor frequência e identificaram pacientes com comprometimento cardiopulmonar mais avançado. Consequentemente, as associações observadas devem ser interpretadas como preditoras de deterioração clínica hospitalar global, e não apenas de mortalidade. Como o choque persistente foi incluído como componente do desfecho primário composto, também desenvolvemos um modelo longitudinal que excluiu o choque cardiogênico na admissão. A consistência dos achados em ambos os modelos multivariáveis reforça a robustez das associações observadas.

### Avaliação longitudinal da disfunção do ventrículo direito

O desempenho discriminativo superior da disfunção persistente do VD na angioTC pulmonar de seguimento (χ^2^ = 15,48), em comparação com a avaliação basal por angioTC pulmonar (p = 0,302), sustenta a hipótese de que a avaliação seriada da função do VD fornece informações prognósticas incrementais em relação à avaliação inicial. Esse achado é consistente com evidências de metanálises que demonstram um aumento aproximado de 2,5 vezes na mortalidade entre pacientes com razão basal VD/VE ≥ 1,0.^6^ A associação mais forte observada para a angioTC pulmonar de seguimento em nossa coorte pode refletir a menor variabilidade interobservador das medidas obtidas por tomografia, a maior padronização da razão VD/VE e a capacidade da avaliação seriada por imagem de identificar a falha na recuperação do VD ao longo do tempo.

A disfunção do VD avaliada por ecocardiografia também esteve significativamente associada ao desfecho primário composto (χ^2^ = 11,18; p < 0,001).^7^ O menor valor do qui-quadrado em comparação com a angioTC pulmonar de seguimento pode ser atribuído à maior dependência do operador na ecocardiografia e à variabilidade de sua disponibilidade entre os centros participantes.^24-27^

Embora a angioTC pulmonar de seguimento tenha demonstrado a associação prognóstica mais forte nesta coorte, esse achado deve ser interpretado no contexto da prática clínica rotineira. A repetição da angioTC pulmonar não era obrigatória pelo protocolo do estudo e foi frequentemente realizada para investigar sintomas persistentes, avaliar disfunção residual do VD ou esclarecer a resposta ao tratamento. Consequentemente, os pacientes submetidos ao exame de imagem de seguimento provavelmente representaram um subgrupo clinicamente selecionado, com maior probabilidade de doença residual e desfechos adversos. Portanto, nossos resultados não devem ser interpretados como evidência de que a angioTC pulmonar seriada seja superior ao acompanhamento ecocardiográfico. Em vez disso, eles sustentam o conceito mais amplo de que a persistência da disfunção do VD detectada durante a hospitalização, independentemente da modalidade de imagem utilizada, carrega importantes informações prognósticas.

Em conjunto, esses achados reforçam o conceito de que a estratificação de risco na EP aguda não deve ser considerada uma avaliação única realizada na admissão hospitalar. Em vez disso, o risco do paciente parece evoluir de acordo com a trajetória de recuperação do VD e da estabilização hemodinâmica. A classificação inicial de risco define a gravidade basal da doença, enquanto a avaliação seriada identifica os pacientes que permanecem em maior risco apesar de receberem tratamento adequado.

### Calibração do modelo

O teste de qualidade de ajuste de Hosmer-Lemeshow para o Modelo 2 apresentou valor de χ^2^ = 7,00 (gl = 6; p = 0,321), indicando calibração satisfatória na coorte do estudo (Figura 4 e Tabela 8).^21^ Esse resultado sustenta a confiabilidade dos riscos preditos pelo modelo ao longo da faixa observada de probabilidades do desfecho. No entanto, a validação externa em uma coorte independente é necessária antes que o modelo possa ser considerado para aplicação clínica rotineira.^18^

**Tabela 8:** – Validação interna (*bootstrap*)

Métrica de desempenho do modelo	Valor
**Desempenho aparente do modelo**	
Pseudo-R^2^ de McFadden	0,381
χ^2^ de Hosmer-Lemeshow (valor de p)	7 (0,321)
AUC-ROC aparente	0,874
**Validação interna por bootstrap (1.000 reamostragens)**	
AUC-ROC ajustada para otimismo	0,852
Inclinação de calibração ajustada para otimismo	0,860
Intercepto de calibração ajustado para otimismo	0,040

AUC-ROC: área sob a curva característica de operação do receptor.

### Evolução hemodinâmica e pressão arterial sistólica

A PAS de seguimento emergiu como um preditor contínuo, estatisticamente significativo e independente do desfecho primário composto (teste *U* de Mann-Whitney, p < 0,001; OR multivariável de 0,90 por mmHg). Cada redução de 1 mmHg na PAS de seguimento pode refletir a progressão de insuficiência compensada para insuficiência descompensada do VD, em consonância com a fisiopatologia do *cor pulmonale* agudo, na qual mesmo reduções discretas da PAS em pacientes inicialmente normotensos podem sinalizar desacoplamento progressivo entre VD e VE e iminente colapso circulatório.^25^ Assim, a persistência da disfunção do VD combinada com valores mais baixos de PAS de seguimento identifica um fenótipo de recuperação fisiológica incompleta. Essa avaliação dinâmica complementa, e não substitui, os sistemas estabelecidos de estratificação de risco baseados na admissão.

### Dados ausentes informativos e suas implicações

A análise de dados ausentes demonstrou que os pacientes sem angioTC pulmonar de seguimento apresentavam gravidade substancialmente maior na admissão, incluindo concentração mediana de NT-proBNP aproximadamente quatro vezes mais elevada (4.100 versus 924 pg/mL; p = 0,001) e escore mediano de PESI aproximadamente 25 pontos superior (125 versus 100; p < 0,001). Esse padrão é consistente com um mecanismo de MNAR, conforme originalmente descrito por Rubin,^22^ indicando que a análise de casos completos no Modelo 2 pode ter produzido estimativas enviesadas, embora a direção e a magnitude desse viés permaneçam incertas.^22,23^

É importante destacar que o padrão observado de MNAR pode ter influenciado tanto a estimativa do tamanho do efeito quanto a seleção dos pacientes. Pacientes que não puderam ser submetidos à angioTC pulmonar de seguimento devido à deterioração clínica precoce podem ter representado um subgrupo com disfunção persistente do VD e piores desfechos que não foi capturado na análise de casos completos. Por outro lado, pacientes que apresentaram rápida melhora clínica também podem ter sido menos propensos a realizar repetição do exame de imagem, uma vez que uma avaliação adicional por angioTC pulmonar foi considerada desnecessária pelos médicos assistentes. Consequentemente, a associação observada entre disfunção persistente do VD e desfechos adversos deve ser interpretada com a devida cautela. Além disso, como a repetição da angioTC pulmonar foi realizada a critério dos médicos assistentes, a decisão de solicitar o exame de seguimento pode ter sido influenciada pela evolução clínica do paciente ou pelos achados ecocardiográficos, introduzindo uma fonte adicional de viés de seleção pós-basal.

Estudos futuros devem incorporar exames seriados de imagem previstos em protocolo, independentemente da evolução clínica, ou aplicar abordagens estatísticas especificamente desenvolvidas para lidar com dados ausentes informativos, incluindo imputação múltipla e análises de sensibilidade apropriadas para mecanismos de MNAR.

### Estratégias de reperfusão e confundimento por indicação

O manejo da EP de risco intermediário-alto e alto evoluiu substancialmente com a crescente adoção de terapias de reperfusão por cateter.^28-33^ O estudo PEITHO^5^ demonstrou que a fibrinólise sistêmica reduziu o desfecho composto de morte ou descompensação hemodinâmica em 7 dias (OR 0,44; p = 0,02), embora esse benefício tenha sido acompanhado por maior risco de sangramento maior. Posteriormente, o estudo FLARE^12^ demonstrou melhora significativa da razão VD/VE após trombectomia mecânica com o sistema FlowTriever em pacientes com EP de risco intermediário, e o estudo FLAME^13^ relatou desfechos favoráveis em pacientes selecionados com EP de alto risco tratados com trombectomia mecânica de grande calibre.

Evidências randomizadas mais recentes do estudo PEERLESS^34^ reforçam ainda mais o papel crescente da reperfusão por cateter, demonstrando melhores desfechos clínicos com trombectomia mecânica em comparação com trombólise dirigida por cateter em pacientes selecionados com EP de risco intermediário. Da mesma forma, o estudo STORM-PE^35^ forneceu evidências prospectivas adicionais que sustentam a eficácia e a segurança das estratégias contemporâneas de reperfusão por cateter.

Em conjunto, esses estudos sugerem que o sucesso da reperfusão não deve ser considerado a etapa final da estratificação de risco. Em vez disso, o uso crescente de terapias avançadas de reperfusão reforça a importância da avaliação contínua da recuperação do VD e da estabilização hemodinâmica durante a hospitalização. Os achados do registro RED-EP sustentam um paradigma de estratificação dinâmica de risco, no qual a reavaliação seriada da função do VD pode identificar pacientes que permanecem em maior risco apesar de uma reperfusão aparentemente bem-sucedida.

Esse conceito é ainda reforçado pelas recomendações do The PERT Consortium,^32^ que enfatizam o acompanhamento clínico e por imagem em longo prazo após a EP aguda, particularmente em pacientes com sintomas persistentes, disfunção residual do VD ou evolução clínica complexa.

### Limitações do estudo

Este estudo apresenta várias limitações. Primeiro, o tamanho relativamente pequeno da amostra (n = 151), particularmente o número limitado de pacientes com dados longitudinais completos de angioTC pulmonar (n = 60), restringiu a complexidade das análises multivariáveis e limitou o poder estatístico. Embora o desempenho do modelo tenha sido inicialmente avaliado por estimativas aparentes, a reamostragem *bootstrap* com 1.000 iterações produziu métricas de desempenho ajustadas para otimismo que sustentaram a estabilidade e a validade interna do modelo longitudinal.^18^

Segundo, a análise de dados ausentes demonstrou um mecanismo informativo de MNAR, com os pacientes sem angioTC pulmonar de seguimento apresentando, em geral, maior gravidade da doença na admissão. Consequentemente, a análise de casos completos pode ter produzido estimativas enviesadas, cuja direção e magnitude permanecem incertas.^22,23^

Terceiro, a angioTC pulmonar de seguimento não era obrigatória pelo protocolo do estudo e foi mais frequentemente realizada em pacientes com sintomas persistentes, recuperação clínica incompleta ou alterações ecocardiográficas persistentes. Portanto, o desempenho prognóstico superior observado para a angioTC pulmonar seriada pode refletir parcialmente a seleção clínica dos pacientes, e não uma vantagem intrínseca da modalidade de imagem.

Por fim, a heterogeneidade terapêutica entre os centros participantes, juntamente com as mudanças nas práticas de tratamento ao longo do período do estudo, limita a interpretação e a generalização das comparações entre as estratégias de reperfusão. Além disso, as análises de acordo com o subtipo de dispositivo de trombectomia foram limitadas pelo pequeno número de pacientes em cada subgrupo.

Consequentemente, esses achados devem ser considerados geradores de hipóteses e necessitam de confirmação em estudos prospectivos de maior porte que incorporem exames seriados de imagem previstos em protocolo e validação externa.

## Conclusões

A reavaliação de marcadores de risco estabelecidos durante a hospitalização pode auxiliar na identificação de pacientes que permanecem em maior risco apesar da classificação inicial de risco e do tratamento. Esses achados sustentam o conceito de estratificação dinâmica de risco na EP aguda, sugerindo que a trajetória de recuperação do VD e da estabilização hemodinâmica fornece informações prognósticas clinicamente relevantes além daquelas obtidas na avaliação inicial. Estudos prospectivos adicionais são necessários para estabelecer a abordagem ideal para a avaliação longitudinal do risco e validar o papel da avaliação seriada da função do VD na prática clínica rotineira.
