# Supplementary material for: Disfunção Persistente do Ventrículo Direito e Evolução Hemodinâmica Predizem Deterioração Hospitalar na Embolia Pulmonar de Risco Intermediário-Alto e Alto
Source: Arq Bras Cardiol. 2026 Jul 31;123(7):e20260468. [Article in Portuguese] doi: 10.36660/abc.20260468 (PMC13668445; doi:10.36660/abc.20260468)
Supplement: Translation

# Translation: Persistent Right Ventricular Dysfunction and Hemodynamic Evolution Predict In-Hospital Deterioration in Intermediate-High- and High-Risk Pulmonary Embolism

**DOI:** 10.36660/abc.20260468i

**Authors:** Miguel A. Quintana, Joaquín Perea, Ricardo Villarreal, Mauricio Soto, Marildes Castro, Rafael Cortês, Patricio Sanhueza, Iván Saavedra, Yhaquelina Quispe, Roger Tenezaca, Roberto Colque, Alexandra Arias, Alex Rivera Toquica, Manuel Álvarez, Ysmenia D. Pérez, Paola Morejón, Karina G. Fernández, Adriana Mogollón, Marcelo Cardona, Gonzalo Emanuel Pérez, Arnaldo López-Ruiz

**Affiliations:** 1 Paraguay Cardiopulomonary Institute LW Randall – Cardiovascular, Asunción – Paraguay; 2 Argentina Sanatorio Guemes – Unidad Coronaria, Buenos Aires – Argentina; 3 Chile Hospital Hernán Henríquez Aravena – Cardiología, Temuco – Chile; 4 Brazil Faculdade de Medicina – Universidade Federal de Minas Gerais – Cardiologia, Belo Horizonte, MG – Brazil; 5 Brazil Hospital Sírio – Libanês Brasília – Cardiologia, Brasília, DF – Brazil; 6 Chile Universidad de Chile – Cardiologia, Santiago – Chile; 7 Brazil Biocor Hospital de Doenças Cardiovasculares Ltda – Cardiologia, Belo Horizonte, MG – Brazil; 8 Bolivia Hospital Obrero No 1, La Paz – Bolivia; 9 Ecuador Hospital de especialidades Guayaquil Abel Gilbert Ponton, Guayaquil – Ecuador; 10 Argentina Sanatorio Allende – Cardiologia, Córdoba – Argentina; 11 Mexico Instituto Nacional de Cardiología Ignacio Chávez, State of Mexico - Mexico; 12 Colombia Clinica los Rosales, Risaralda – Colombia; 13 Colombia Fundacion Santa Fe de Bogota – Cardiologia, Bogotá – Colombia; 14 Dominican Republic Hospital General y Especialidades Dr.Mario Tolentino Dipp – Cardiologia, Santo Domingo – Dominican Republic; 15 Ecuador Clinica Guayaquil – Cardiologia, Guayaquil – Ecuador; 16 Peru Hospital de Emergencias José Casimiro Ulloa – Cardiologia, Lima – Peru; 17 Venezuela Centro Medico San Francisco – Cardiologia, Caracas – Venezuela; 18 Argentina Hola Hospital Privado de Rosario – Cardiologia, Rosario – Argentina; 19 Argentina Clinica Olivos – Cardiology, Buenos Aires – Argentina; 20 USA AdventHealth Orlando -– Department of Critical Care, Orlando, Florida – USA

**Keywords:** Pulmonary Embolism, Right Ventricular Dysfunction, Prognosis, Hemodynamics, Cardiogenic Shock, Reperfusion, Thrombectomy, Logistic Models

## Abstract

**Background:**

Risk stratification in acute pulmonary embolism (PE) is based primarily on findings obtained at hospital admission. However, whether serial assessment of right ventricular (RV) dysfunction and hemodynamic changes provides incremental prognostic value beyond the initial risk stratification remains uncertain.

**Objectives:**

To evaluate longitudinal clinical and imaging variables associated with adverse in-hospital outcomes in patients with intermediate-high- and high-risk acute PE.

**Methods:**

This prospective registry included patients with intermediate-high- and high-risk acute PE. The primary composite endpoint comprised in-hospital mortality, invasive mechanical ventilation, persistent shock or hypotension, and extracorporeal membrane oxygenation. Longitudinal variables were assessed 24-72 hours after admission. All statistical tests were two-tailed, and a p-value < 0.05 was considered statistically significant. Multivariable logistic regression models were constructed, and discriminative performance was evaluated using the area under the receiver operating characteristic curve (AUC-ROC). A prespecified missing-data analysis was performed to assess the availability of follow-up computed tomography pulmonary angiography (CTPA).

**Results:**

The primary composite endpoint occurred in 21.2% of the 151 patients, with an in-hospital mortality rate of 9.3%. Persistent RV dysfunction on follow-up CTPA (χ^2^ = 15.48; p < 0.001) and follow-up systolic blood pressure (SBP; p < 0.001) were the strongest individual predictors of adverse outcomes. In the longitudinal multivariable model (n = 60), persistent RV dysfunction on follow-up CTPA (OR 9.70; 95%CI, 1.96-47.89; p = 0.005) and follow-up SBP (OR 0.90; 95%CI, 0.83-0.96; p = 0.003) remained independent predictors. The model demonstrated excellent discrimination (AUC-ROC = 0.874) and adequate calibration. The missing-data analysis showed that patients without follow-up CTPA were more severely ill at baseline, consistent with a missing not at random mechanism.

**Conclusions:**

Reassessment of established risk markers during hospitalization may improve identification of patients who remain at high risk despite initial risk classification and treatment. Persistent RV dysfunction and lower follow-up SBP were independently associated with adverse in-hospital outcomes, which supports the use of dynamic risk stratification in acute PE.

## Introduction

Acute pulmonary embolism (PE) remains a leading cause of cardiovascular morbidity and mortality, with an estimated global incidence of more than 1-2 cases per 1,000 person-years.^1,2^ Contemporary risk stratification frameworks, including those endorsed by the European Society of Cardiology (ESC) and the American Heart Association/American College of Cardiology (AHA/ACC), classify patients according to hemodynamic status, right ventricular (RV) dysfunction detected by echocardiography or computed tomography pulmonary angiography (CTPA), and cardiac biomarkers, including troponin and B-type natriuretic peptides (BNP).^1^

The Pulmonary Embolism Severity Index (PESI) and its simplified version (sPESI), both derived and prospectively validated in large cohorts, provide reliable estimates of 30-day mortality risk based solely on clinical variables.^3,4^ Nevertheless, a clinically important proportion of patients classified as having intermediate-high- or high-risk PE experiences in-hospital clinical deterioration that is not fully predicted by the initial assessment. The PEITHO trial highlighted the prognostic heterogeneity within the intermediate-risk population, demonstrating that the combination of RV dysfunction and increased troponin levels does not consistently identify patients who will subsequently develop hemodynamic instability.^5^

RV dysfunction, assessed by either echocardiography or CTPA, is an independent predictor of adverse outcomes. Some meta-analyses have shown that echocardiographic RV dysfunction is associated with a 2.29-fold increase in short-term mortality, whereas an RV/left ventricular (LV) ratio ≥ 1.0 on CTPA is associated with an approximately 2.5-fold increase in mortality risk.^6,7^ Cardiac troponins and N-terminal pro-BNP (NT-proBNP) further improve risk stratification in normotensive patients.^8-11^

The concept of dynamic risk stratification, based on serial assessment of hemodynamic parameters, RV function, and cardiac biomarkers during hospitalization, has received increasing attention. Persistent RV dysfunction on follow-up imaging may reflect unresolved pulmonary vascular obstruction, delayed hemodynamic recovery, or progressive circulatory impairment.^1,2^ In parallel, advances in catheter-based reperfusion therapies, including percutaneous mechanical thrombectomy (PMT) with devices such as FlowTriever, evaluated in the FLARE and FLAME studies, and the Penumbra system, have expanded treatment options for patients with high-risk PE. However, in observational studies, the relationship between reperfusion strategy and clinical outcomes remains difficult to interpret because of indication bias.^5,12-14^

The RED-EP registry is a prospective multicenter cohort established to characterize the clinical presentation, therapeutic strategies, and outcomes of patients with intermediate-high- and high-risk PE in real-world Latin American settings. The present analysis investigated whether longitudinal changes in hemodynamic parameters and RV function provide prognostic information beyond admission-based risk assessment, and whether reperfusion strategy is independently associated with in-hospital outcomes after adjustment for disease severity.

## Methods

### Study design and population

This prospective, multicenter, observational cohort study enrolled consecutive patients with CTPA-confirmed acute PE classified as intermediate-high or high risk at participating centers across the Americas over a 12-month period, from April 2025 to April 2026. Data were collected prospectively using the mawe platform. High-risk PE was defined according to the 2019 ESC guidelines as hemodynamic instability, including cardiogenic shock or sustained hypotension (systolic blood pressure [SBP] < 90 mmHg or a decrease of ≥ 40 mmHg lasting more than 15 minutes that could not be explained by new-onset arrhythmia, hypovolemia, or sepsis).^1^ Intermediate-high-risk PE was defined as the presence of both RV dysfunction on imaging (CTPA and/or echocardiography) and increased cardiac biomarkers (troponin and/or BNP or NT-proBNP) in hemodynamically stable patients.^1^

### Study objectives

The primary objective was to evaluate longitudinal variables associated with adverse in-hospital outcomes in patients with intermediate-high- and high-risk acute PE, regardless of whether they underwent reperfusion therapy. Reperfusion strategies were selected according to institutional protocols and local resource availability across participating centers.

The secondary objectives were to i) compare admission and longitudinal variables as predictors of adverse outcomes; ii) evaluate the association between specific reperfusion strategies and clinical outcomes; and iii) perform an exploratory analysis of outcomes according to the subtype of PMT device.

### Variable definitions

Admission variables were age, sex, body weight, height, comorbidities, heart rate, SBP, SpO_2_, PESI,^3^ sPESI,^4^ Registro Informatizado de Enfermedad TromboEmbólica bleeding score, high-sensitivity troponin, NT-proBNP, BNP, D-dimer, baseline RV dysfunction on CTPA (defined as an RV/LV ratio ≥ 1.0 on the axial four-chamber view),^6^ baseline RV dysfunction on echocardiography, and the presence of cardiogenic shock.

Longitudinal variables were assessed 24-72 hours after hospital admission and included follow-up heart rate, SBP, SpO_2_, RV dysfunction on follow-up CTPA and echocardiography, repeat troponin, and repeat NT-proBNP measurements. Follow-up CTPA and echocardiography were performed at the discretion of the treating physicians and were not mandated by the study protocol. Consequently, follow-up imaging was analyzed as a categorical variable (available or unavailable within 24-72 hours after admission) rather than according to the exact timing of image acquisition. The median time to follow-up assessment within this interval was not systematically recorded across participating centers.

Treatment variables were receipt of any reperfusion therapy, advanced reperfusion therapy (systemic thrombolysis, catheter-directed thrombolysis, or PMT), type of thrombectomy device (FlowTriever, Penumbra, pigtail catheter, or other), and use of extracorporeal membrane oxygenation (ECMO).

### Primary composite endpoint

The primary composite endpoint comprised: i) all-cause in-hospital mortality; ii) persistent shock or hypotension requiring vasopressor support; iii) requirement for invasive mechanical ventilation; and iv) ECMO. These components represent a clinical continuum of hemodynamic deterioration in patients with high-risk PE. Persistent shock or hypotension requiring vasopressor support reflects ongoing circulatory failure and is a well-established surrogate marker of mortality in this population, particularly among patients who underwent reperfusion because of severe clinical presentation. Individual components of the composite endpoint were also analyzed as secondary outcomes.^15-17^

The composite endpoint was chosen to capture clinically meaningful manifestations of in-hospital hemodynamic deterioration. Although the individual components differ in clinical severity, they all reflect failure to achieve hemodynamic stabilization after the diagnosis and treatment of acute PE and represent progressive stages of a shared pathophysiological process characterized by persistent RV dysfunction, circulatory compromise, and end-organ hypoperfusion.

### Statistical analysis

#### Normality assessment and descriptive statistics

The distribution of continuous variables was evaluated by visual inspection of histograms and quantile-quantile (Q-Q) plots, and confirmed using the Shapiro-Wilk test. All key continuous variables demonstrated non-normal, right-skewed distributions. Accordingly, continuous variables are presented as the median and IQR (25th-75th percentile). Categorical variables are presented as frequencies and percentages. Missing data are reported for each variable, and no imputation was performed in the primary analyses.

#### Bivariable analysis

Associations between categorical variables and the primary composite endpoint were evaluated using Pearson’s chi-square test of independence. Fisher’s exact test was applied when the expected cell count was < 5. Effect size was quantified using Cramér’s V.

Continuous variables were compared using the Mann-Whitney *U* test (Wilcoxon rank-sum test), given their non-normal distributions. Results are presented as median and IQR (25th-75th percentile). Effect size for continuous variables was calculated using the rank-biserial correlation, defined as $r=1-(2U)/\left(n_{1}\times n_{2}\right)$ . All statistical tests were two-sided, and a p-value < 0.05 was considered statistically significant.

#### Multivariate logistic regression

Two multivariable logistic regression models were fitted using maximum likelihood estimation. The logit link function was used to model the log odds of the primary outcome according to the equation: $\mathrm{logit}(P)=\beta_{0}+\beta_{1}X_{1}+\beta_{2}X_{2}+\ldots +\beta_{k}X_{k}$ . Odds ratios (ORs) and corresponding 95%CIs were obtained by exponentiating the regression coefficients, and statistical significance was assessed using the Wald test.^18^

Model 1 (admission model) included cardiogenic shock at admission, follow-up RV dysfunction on echocardiography, follow-up SBP, and reperfusion strategy (n = 106 complete cases). Model 2 (longitudinal model) included persistent RV dysfunction on follow-up CTPA, follow-up SBP, and reperfusion strategy (n = 60 complete cases). Admission cardiogenic shock was intentionally excluded from Model 2 to avoid potential collider bias arising from the inclusion of in-hospital shock as a component of the primary composite endpoint.

Variables considered clinically relevant and/or associated with the primary endpoint in bivariable analyses (p < 0.10) were considered for inclusion in the multivariable models. Final model selection also accounted for the limited number of outcome events to reduce the risk of overfitting. To minimize multicollinearity and redundancy, variables representing overlapping clinical constructs were not entered simultaneously into the same model, for example, SBP and cardiogenic shock, or multiple biomarkers reflecting myocardial injury or cardiac stress.^19-21^

#### Model performance and calibration

Overall model performance was evaluated using the likelihood ratio test, McFadden’s pseudo-R^2^, and the Akaike information criterion. A McFadden’s pseudo-R^2^ value ≥ 0.20 was considered indicative of excellent model fit. Discriminative performance was assessed using the area under the receiver operating characteristic curve (AUC-ROC).

Model calibration was evaluated using the Hosmer-Lemeshow goodness-of-fit test. To assess potential overfitting and optimism in the apparent model performance, internal validation was performed by bootstrap resampling with 1,000 iterations. Optimism-adjusted estimates of the AUC-ROC, calibration slope, and calibration intercept were calculated to evaluate model stability and the expected reliability of risk predictions in independent populations.

#### Missing-data analysis

Follow-up CTPA was available for 72 of the 151 patients (47.7%). A prespecified missing-data analysis compared baseline characteristics between patients with and without follow-up CTPA to investigate whether the missing-data mechanism was consistent with missing completely at random, missing at random, or missing not at random (MNAR).^22,23^ Continuous variables were compared using the Mann-Whitney *U* test, and categorical variables using Pearson’s chi-square test.

#### Reperfusion subgroup analysis

Baseline characteristics and clinical outcomes were compared descriptively across reperfusion strategy groups (no reperfusion, systemic thrombolysis, and PMT) to characterize potential confounding by indication.^5,12,13^ An exploratory comparison was also performed according to the thrombectomy device used (FlowTriever, Penumbra, or pigtail catheter).

#### Software

All statistical analyses were performed using JASP version 0.97.0. Nonparametric tests, logistic regression analyses, and ROC curve analyses were conducted in JASP. Confirmatory analyses and figure generation were performed in Python 3.12 using Matplotlib version 3.8.0. Artificial intelligence-assisted code generation was used during the programming process, and all code, analyses, and outputs were independently reviewed and verified by the authors.

## Results

### Cohort characteristics

A total of 151 patients were included in the analysis. The primary composite endpoint occurred in 32 patients (21.2%), and the in-hospital mortality rate was 9.3% (n = 14). Baseline characteristics, longitudinal variables, and treatment data, stratified according to the occurrence of the primary composite endpoint, are presented in Table 1 and Figure 1.

**Table 1:** – Baseline characteristics, follow-up variables, and clinical outcomes according to the primary composite endpoint

Variable	Overall (n = 151)	No event (n = 119)	Event (n = 32)	p-value
**Demographics and clinical scores**				
Age, median (IQR), years	67 (57-76)	66 (57-76)	68 (58-75)	0.440
Male sex, n (%)	67 (44.4)	55 (46.2)	12 (37.5)	0.378
PESI score, median (IQR)	110 (79-138)	105 (77-136)	124 (89-158)	0.061
sPESI score, median (IQR)	2 (1-3)	2 (1-3)	3 (1-3)	0.001
High-risk PE (cardiogenic shock), n (%)	19 (12.6)	7 (5.9)	12 (37.5)	< 0.001
**Baseline imaging and biomarkers**				
RV dysfunction on echocardiography, n (%)	93 (62.0)	70 (63.1)	23 (71.9)	0.357
RV/LV ratio > 1.0 on CTPA, n (%)	87 (57.6)	65 (54.6)	22 (68.8)	0.151
Troponin, median (IQR), ng/L	98 (35-188)	91 (35-188)	112 (40-189)	0.347
NT-proBNP, median (IQR), pg/mL	2.812 (680-5.130)	2.450 (675-4.515)	3.927 (1.063-7.562)	0.105
D-dimer, median (IQR), µg/L	9.800 (4.270-14.023)	9.800 (4.270-14.023)	7.525 (1.834-11.765)	0.126
**Treatment strategies**				
Any reperfusion therapy, n (%)	78 (51.7)	61 (51.3)	17 (53.1)	0.851
Advanced reperfusion therapy, n (%)	71 (47.0)	58 (48.7)	13 (40.6)	0.414
PMT, n (%)	46 (36.8)	44/99 (44.4)	2/18 (11.1)	0.040
Systemic thrombolysis, n (%)	24 (15.9)	17 (14.3)	7 (21.9)	0.113
**Follow-up variables (24-72 h)**				
Follow-up SBP, median (IQR), mmHg	—	125 (115-130)	103 (100-127)	< 0.001
Persistent RV dysfunction on follow-up CTPA, n (%)	22/72 (30.6)	9/50 (18.0)	13/22 (59.1)	< 0.001
Persistent RV dysfunction on follow-up echocardiography, n (%)	39/114 (34.2)	23/75 (30.7)	16/39 (41.0)	0.001
Follow-up NT-proBNP, median (IQR), pg/mL	—	656 (197-1.678)	1.740 (920-7.555)	0.039
**Clinical outcomes**				
In-hospital mortality, n (%)	14 (9.3)	0 (0)	14 (43.8)	N/A
Shock or hypotension requiring vasopressors, n (%)	22 (14.6)	0 (0)	22 (68.8)	N/A
Invasive mechanical ventilation, n (%)	6 (4.0)	0 (0)	6 (18.8)	N/A
ECMO, n (%)	7 (4.6)	0 (0)	7 (21.9)	N/A
Primary composite endpoint, n (%)	32 (21.2)	0 (0)	32 (100)	N/A

CTPA: computed tomography pulmonary angiography; ECMO: extracorporeal membrane oxygenation; LV: left ventricle; N/A: not applicable; NT-proBNP: N-terminal pro-B-type natriuretic peptide; PE: pulmonary embolism; PESI: Pulmonary Embolism Severity Index; PMT: percutaneous mechanical thrombectomy; RV: right ventricle; SBP: systolic blood pressure; sPESI: simplified PESI. Continuous variables are presented as the median (IQR [25th-75th percentile]), and categorical variables as n (%). Continuous variables were compared using the Mann-Whitney U test, whereas categorical variables were compared using Pearson’s chi-square test or Fisher’s exact test, as appropriate. Denominators for follow-up variables vary according to data availability: follow-up CTPA was available for 72 of 151 patients (47.7%), and follow-up echocardiography for 114 of 151 patients (75.5%). The denominator for PMT reflects the number of patients with available procedural data (n = 125).

**Figure 1:** – Baseline clinical and biomarker characteristics according to the primary composite endpoint. Boxplots comparing baseline clinical, hemodynamic, and biomarker variables between patients who experienced the primary composite endpoint (red) and those who did not (blue) (n = 151). Continuous variables were compared using the two-sided Mann-Whitney U test. Boxes represent the IQR, the horizontal line indicates the median, whiskers extend to 1.5 × IQR, and points denote outliers. ***p < 0.001; **p < 0.01; *p < 0.05. NT-proBNP: N-terminal pro-B-type natriuretic peptide; NS: not significant; PESI: Pulmonary Embolism Severity Index; SBP: systolic blood pressure.

### Bivariable analysis of the primary composite endpoint

The complete bivariable analysis is presented in Table 2, Table 3 and Table 4 and the bivariate quantitative analysis is shown in Figure 2. Among the imaging variables, persistent RV dysfunction on follow-up CTPA was the strongest categorical predictor of the primary composite endpoint (χ^2^ = 15.48, degrees of freedom [df] = 1, p < 0.001; OR 6.95).^6,7^ Among the continuous variables, follow-up SBP showed the strongest association with adverse outcomes (Mann-Whitney *U* test, p < 0.001).

**Table 2:** – Baseline characteristics according to reperfusion status

Variable	No reperfusion (n=73)	Reperfusion (n=78)	p-value
Age, years, median (IQR)	69 (57-79)	65 (57-73)	0.574
Body weight, kg, median (IQR)	75 (68-83)	84 (75-95)	0.336
Height, cm, median (IQR)	168 (160-172)	170 (165-176)	0.015^*^
PESI score, median (IQR)	100 (70-123)	126 (90-150)	0.040^*^
sPESI score, median (IQR)	2 (1-3)	2 (1-3)	0.333
Heart rate, beats/min, median (IQR)	90 (80-110)	118 (96-125)	0.237
SBP, mmHg, median (IQR)	120 (110-135)	120 (109-135)	0.328
SpO2, %, median (IQR)	92 (89-95)	86 (84-90)	0.165
Persistent RV dysfunction on follow-up CTPA, n (%)	7/50 (14.0)	13/22 (59.1)	< 0.001^***^
Cardiogenic shock at admission, n (%)	20/132 (15.1)	12/19 (63.2)	< 0.001^***^
Primary composite endpoint, n (%)	15 (20.5)	17 (21.8)	0.851
In-hospital mortality, n (%)	8 (11.0)	6 (7.7)	0.489

CTPA: computed tomography pulmonary angiography; PESI: Pulmonary Embolism Severity Index; RV: right ventricle; SBP: systolic blood pressure; sPESI: simplified PESI. Continuous variables are presented as the median (IQR [25th-75th percentile]), and categorical variables as n (%). Continuous variables were compared using the two-sided Mann-Whitney U test, and categorical variables using Pearson’s chi-square test. *p < 0.05; ***p < 0.001. The denominator for persistent RV dysfunction on follow-up CTPA reflects the number of patients who underwent repeat CTPA imaging.

**Table 3:** – Bivariable analysis of qualitative variables associated with the primary composite endpoint

Variable	No event (n = 119)	Event (n = 32)	p-value	OR (95%CI)
Baseline RV dysfunction on echocardiography, n (%)	70 (63.1)	23 (71.9)	0.357	—
Baseline RV/LV ratio >1.0 on CTPA, n (%)	65 (54.6)	22 (68.8)	0.151	—
Any reperfusion therapy, n (%)	58 (48.7)	15 (46.9)	0.851	0.93 (0.43-2.01)
Cardiogenic shock at admission, n (%)	7 (5.9)	12 (37.5)	< 0.001^***^	9.60 (3.47-26.57)
Persistent RV dysfunction on follow-up CTPA, n (%)^†^	9 (18.0)	13 (59.1)	< 0.001^***^	6.95 (2.39-20.21)
Persistent RV dysfunction on follow-up echocardiography, n (%)^‡^	23 (30.7)	16 (61.5)	< 0.001^***^	3.62 (1.48-8.86)

CTPA: computed tomography pulmonary angiography; df: degrees of freedom; LV: left ventricle; OR: odds ratio; RV: right ventricle. Data are presented as n (%). p-values were calculated using Pearson’s chi-square test (df = 1, two-sided). Fisher’s exact test was used when the expected cell count was < 5. †Follow-up CTPA available for 72 patients. ‡Follow-up echocardiography available for 114 patients. ***p < 0.001.

**Table 4:** – Bivariable analysis of quantitative variables associated with the primary composite endpoint

Variable	No Event (n=119)	Event (n=32)	p-value
Height, cm, median (IQR)	170 (162-176)	165 (162-170)	0.015^*^
Follow-up SBP, mmHg, median (IQR)	124 (115-130)	102 (100-127)	< 0.001^***^
Follow-up SpO2, %, median (IQR)	95 (93-96)	94 (92-95)	0.013^*^
Troponin, ng/L, median (IQR)	91 (35-188)	112 (40-188)	0.014^*^
Follow-up NT-proBNP, pg/mL, median (IQR)	656 (197-1.678)	1.740 (920-7.555)	0.039^*^

NT-proBNP: N-terminal pro-B-type natriuretic peptide; SBP: systolic blood pressure. Continuous variables are presented as the median (IQR [25th-75th percentile]). Group comparisons were performed using the two-sided Mann-Whitney U test. *p < 0.05; ***p < 0.001.

**Figure 2:** – Distribution of key continuous variables according to the primary composite endpoint. Boxplots comparing baseline and follow-up (24-72 hours) continuous variables between patients who experienced the primary composite endpoint (red) and those who did not (blue). Follow-up SBP and follow-up NT-proBNP were significantly associated with the primary composite endpoint. Boxes represent the IQR, the horizontal line indicates the median, whiskers extend to 1.5 × IQR, and points denote outliers. Statistical comparisons were performed using the two-sided Mann-Whitney U test. ***p < 0.001; *p < 0.05. Sample sizes are shown below each boxplot. NS: not significant; NT-proBNP: N-terminal pro-B-type natriuretic peptide; PESI: Pulmonary Embolism Severity Index; SBP: systolic blood pressure.

No reperfusion strategy was significantly associated with the primary composite endpoint in the bivariable analyses. Baseline imaging findings and cardiac biomarkers were generally not significantly associated with adverse outcomes in this cohort, whereas several longitudinal variables demonstrated stronger and more consistent associations with the primary composite endpoint.^8-11^

### Multivariate logistic regression

Two multivariable logistic regression models were developed (Table 5 and Figure 3). In both models, follow-up SBP remained an independent predictor of the primary composite endpoint. In the longitudinal model (Model 2), persistent RV dysfunction on follow-up CTPA also remained independently associated with adverse in-hospital outcomes.

**Table 5:** – Multivariable logistic regression analysis of independent predictors of the primary composite endpoint

Variable	β coefficient	SE	Wald z statistic	OR (95%CI)	p-value
**Model 1 – Admission model**					
n = 106; McFadden’s pseudo-R^2^ = 0.301; AUC; ROC = 0.807; LR χ2 = 35.54; LR p < 0.001; AIC = 92.6					
Intercept	6.64	2.69	2.47	765.7 (N/A)	0.014
Cardiogenic shock at admission	2.38	0.75	3.19	10.82 (2.51-46.69)	0.001^**^
Persistent RV dysfunction on follow-up echocardiography	0.73	0.69	1.07	2.08 (0.54-7.98)	0.286
Follow-up SBP (per 1 mmHg)	–0.071	0.023	–3.06	0.93 (0.89-0.97)	0.002^**^
Reperfusion strategy	–0.80	0.66	–1.22	0.45 (0.12-1.63)	0.224
**Model 2 – Longitudinal model**					
n = 60; McFadden’s pseudo-R^2^ = 0.381; AUC-ROC = 0.874; Hosmer-Lemeshow χ^2^ = 7.00 (df = 6, p = 0.321); LR χ^2^ = 27.96; LR p < 0.001; AIC = 53.3					
Intercept	10.61	4.13	2.57	N/A	0.010
Persistent RV dysfunction on follow-up CTPA	2.27	0.81	2.79	9.70 (1.96-47.89)	0.005^**^
Follow-up SBP (per 1 mmHg)	–0.108	0.037	–2.95	0.90 (0.83-0.96)	0.003^**^
Reperfusion strategy	–0.17	0.80	–0.21	0.85 (0.18-4.05)	0.836

AIC: Akaike information criterion; AUC-ROC: area under the receiver operating characteristic curve; CTPA: computed tomography pulmonary angiography; df: degrees of freedom; LR: likelihood ratio; N/A: not applicable; OR: odds ratio; RV: right ventricle; SBP: systolic blood pressure; SE: standard error. Multivariable logistic regression models were fitted using maximum likelihood estimation. ORs were obtained by exponentiating the regression coefficients (OR = exp[β]), and 95%CIs were calculated as exp(β ± 1.96 × SE). Statistical significance was assessed using the Wald test (z = β/SE). McFadden’s pseudo-R2 values ≥ 0.20 were considered indicative of excellent model fit. Calibration was assessed using the Hosmer-Lemeshow goodness-of-fit test with eight groups (df = 6); p > 0.05 indicates adequate calibration. Discriminative performance was evaluated using the AUC-ROC based on apparent (in-sample) predictions. **p < 0.01.

**Figure 3:** – Forest plot of the longitudinal multivariable logistic regression model (Model 2). Forest plot showing the adjusted ORs and 95%CIs for the independent predictors of the primary composite endpoint identified in the longitudinal multivariable logistic regression model. The model was fitted using maximum likelihood estimation (n = 60 complete cases). ORs were calculated as exp(β), and 95%CIs as exp(β ± 1.96 × SE). The vertical reference line indicates an OR of 1.0. Red diamonds denote statistically significant risk factors (OR > 1, p < 0.05), blue diamonds denote statistically significant protective factors (OR < 1, p < 0.05), and gray diamonds denote nonsignificant associations. Model performance: AUC-ROC = 0.874; McFadden’s pseudo-R2 = 0.381; Hosmer-Lemeshow p = 0.321; LR p < 0.001 . AUC-ROC: area under the receiver operating characteristic curve; CTPA: computed tomography pulmonary angiography; LR: likelihood ratio; NS: not significant; OR: odds ratio; RV: right ventricle; SBP: systolic blood pressure; SE: standard error.

Overall, the longitudinal model demonstrated superior discriminative ability and calibration compared with the admission model. The apparent AUC-ROC was 0.874, whereas the optimism-adjusted AUC obtained by bootstrap validation was 0.852 (Figure 4). Calibration remained satisfactory after internal validation, with an adjusted calibration slope of 0.86 and an adjusted calibration intercept of 0.04, which indicates good agreement between predicted and observed risks and minimal evidence of systematic prediction bias.

**Figure 4:** – Discrimination and calibration of the longitudinal prediction model. Panel A: ROC curves comparing the longitudinal model (Model 2; AUC-ROC = 0.874, solid red line) with the admission model (Model 1; AUC-ROC = 0.807, dashed blue line). Panel B: Calibration plot for the longitudinal model. Each point represents one risk decile, with point size proportional to the number of patients in the corresponding group. The Hosmer-Lemeshow goodness-of-fit test showed adequate calibration (χ2 = 7.00, df = 6, p = 0.321). The bootstrap-adjusted AUC-ROC was 0.852, which supports the robustness of the model’s discriminative performance. AUC-ROC: area under the receiver operating characteristic curve; df: degrees of freedom.

### Missing-data analysis of follow-up computed tomography availability

Follow-up CTPA was available for 72 of the 151 patients (47.7%). Compared with patients who underwent repeat imaging, those without follow-up CTPA generally had greater disease severity at baseline (Table 6 and Figure 5), which suggests an informative missing-data mechanism consistent with MNAR.^22,23^ These findings indicate that the decision to perform follow-up imaging was influenced by the patient’s clinical course rather than occurring randomly. Rates of cardiogenic shock at admission, the primary composite endpoint, and reperfusion therapy did not differ significantly between groups.

**Table 6:** – Missing-data analysis according to follow-up CTPA availability

Variable	Follow-up CTPA available (n = 72)	Follow-up CTPA not available (n = 79)	p-value
**Continuous variables**			
Age, years, median (IQR)	68 (56-76)	66 (58-72)	0.912
PESI score, median (IQR)	100 (53-124)	125 (100-150)	< 0.001^***^
sPESI score, median (IQR)	2 (1-3)	2 (1-3)	0.010^*^
Admission SBP, mmHg, median (IQR)	118 (105-130)	126 (110-138)	0.022^*^
**Admission heart rate, beats/min, median (IQR)**	92 (80-115)	110 (100-122)	0.001^***^
**Admission SpO_**2**_, %, median (IQR)**	90 (86-94)	87 (85-92)	0.018^*^
**Troponin, ng/L, median (IQR)**	52 (13-174)	134 (73-188)	0.002^**^
**NT-proBNP, pg/mL, median (IQR)**	924 (98-3.814)	4.100 (1.895-5.800)	0.001^***^
**Categorical variables**			
Cardiogenic shock at admission, n (%)	12 (16.7)	7 (8.9)	0.231
Primary composite endpoint, n (%)	20 (27.8)	12 (15.2)	0.091
Any reperfusion therapy, n (%)	34 (47.2)	44 (55.7)	0.380
Baseline RV dysfunction on echocardiography, n (%)	39 (54.2)	55 (69.2)	0.084

CT: computed tomography; NT-proBNP: N-terminal pro-B-type natriuretic peptide; PESI: Pulmonary Embolism Severity Index; RV: right ventricle; SBP: systolic blood pressure; sPESI: simplified PESI. Continuous variables are presented as the median (IQR [25th-75th percentile]), and categorical variables as n (%). Continuous variables were compared using the two-sided Mann-Whitney U test, whereas categorical variables were compared using Pearson’s chi-square test. ***p < 0.001; **p < 0.01; *p < 0.05.

**Figure 5:** – Baseline characteristics according to follow-up CTPA availability. Boxplots comparing baseline clinical and biomarker variables between patients with follow-up CTPA available (blue, n = 72) and those without follow-up CTPA (orange, n = 79). Patients without follow-up CTPA had higher baseline PESI scores, heart rate, troponin, and NT-proBNP levels, consistent with an informative missing-data pattern compatible with MNAR. Boxes represent the IQR, the horizontal line indicates the median, whiskers extend to 1.5 × IQR, and points denote outliers. Statistical comparisons were performed using the two-sided Mann-Whitney U test. ***p<0.001; **p<0.01; *p<0.05. CTPA: computed tomography pulmonary angiography; MNAR: missing not at random; NT-proBNP: N-terminal pro-B-type natriuretic peptide; PESI: Pulmonary Embolism Severity Index; SBP: systolic blood pressure.

The observed MNAR pattern is likely explained by two competing mechanisms. First, some patients experienced early hemodynamic deterioration and were too clinically unstable to undergo repeat imaging. Second, patients with rapid clinical improvement may not have undergone follow-up CTPA because additional imaging was considered unnecessary by the treating physicians. Together, these mechanisms may have enriched the analyzed sample with patients of intermediate clinical severity. Consequently, the direction and magnitude of potential bias in the estimates derived from Model 2 cannot be determined without formal missing-data methods, such as multiple imputation.^22,23^ These findings underscore the need for prospective multicenter studies incorporating protocol-mandated follow-up imaging at predefined time points, regardless of clinical trajectory, to better define the prognostic value of persistent RV dysfunction and reduce potential selection bias related to repeat imaging.

### In-hospital mortality

The in-hospital mortality rate was 9.3% (n=14). In the bivariable analysis, mortality was significantly associated with cardiogenic shock at admission (χ^2^ = 7.51, p = 0.006), persistent shock or hypotension requiring vasopressor support during hospitalization (χ^2^ = 5.54, p = 0.019), RV dysfunction on echocardiography (χ^2^ = 4.57, p = 0.032), and the need for invasive mechanical ventilation (χ^2^ = 4.30, p = 0.038). In contrast, neither the baseline RV/LV ratio on CTPA (p = 0.970) nor baseline troponin levels (p = 0.851) was significantly associated with in-hospital mortality, further supporting the greater prognostic value of longitudinal variables compared with admission-based measures.^6,8^

### Reperfusion strategies

Among the 78 patients (51.7%) who underwent reperfusion therapy, 24 (15.9%) received systemic thrombolysis and 46 underwent PMT, most commonly with the FlowTriever device (n = 36). Neither reperfusion therapy overall (p = 0.851) nor advanced reperfusion therapy (p = 0.414) was independently associated with the primary composite endpoint.^5,12,13^

As shown in Table 7, the distribution of baseline characteristics across treatment groups reflects substantial confounding by indication. Patients treated with systemic thrombolysis had the highest prevalence of cardiogenic shock at admission (37.5%), whereas those undergoing mechanical thrombectomy had higher PESI scores and NT-proBNP concentrations but a lower prevalence of cardiogenic shock (13.0%), consistent with a hemodynamically stable yet high-risk clinical phenotype. The numerically lower incidence of the primary composite endpoint in the thrombectomy group (13.0% versus 20.5% in the no-reperfusion group) should therefore be interpreted with caution, as it is likely attributable to treatment selection based on baseline clinical characteristics rather than to a true treatment effect.

**Table 7:** – Baseline characteristics and clinical outcomes according to reperfusion strategy

Variable	No reperfusion (n = 73)	Systemic thrombolysis (n = 24)	PMT (n = 46)
Cardiogenic shock at admission, n (%)	3 (4.1)	9 (37.5)	6 (13.0)
PESI score, median (IQR)	100 (70-123)	117 (105-148)	137 (116-156)
NT-proBNP, pg/mL, median (IQR)	820 (86-5.805)	3.128 (1.554-6.852)	3.828 (2.201-4.912)
Primary composite endpoint, n (%)	15/73 (20.5)	8/24 (33.3)	6/46 (13.0)
In-hospital mortality, n (%)	8/73 (11.0)	3/24 (12.5)	2/46 (4.3)

NT-proBNP: N-terminal pro-B-type natriuretic peptide; PESI: Pulmonary Embolism Severity Index; PMT: percutaneous mechanical thrombectomy. Data are presented descriptively. No formal statistical comparisons were performed because of the small sample sizes and the presence of substantial confounding by indication. Reperfusion groups were not mutually exclusive for all patients.

## Discussion

### Principal findings

The present findings suggest that risk markers already incorporated into contemporary ESC and AHA/ACC risk stratification frameworks retain substantial prognostic value when reassessed during hospitalization. Serial evaluation of RV recovery and hemodynamic stabilization appears to provide incremental prognostic information beyond that available at admission. Persistent RV dysfunction on follow-up CTPA (OR 9.70; 95%CI, 1.96-47.89; p = 0.005) and follow-up SBP (OR 0.90 per mmHg; 95%CI, 0.83-0.96; p = 0.003) emerged as the principal independent predictors of adverse in-hospital outcomes, whereas admission biomarkers, established risk scores, and reperfusion strategies were not independently associated with the primary composite endpoint. These findings expand the prognostic framework proposed by the 2019 ESC guidelines and the 2026 AHA/ACC guideline by highlighting the importance of dynamic risk assessment during hospitalization.^1,14^ As shown in Figure 6 and the Central Illustration, patients with persistent RV dysfunction accompanied by unfavorable hemodynamic evolution experienced substantially higher rates of in-hospital clinical deterioration.

**Figure 6:** – Statistical significance of variables associated with the primary composite endpoint. Bar lengths represent –log10 (p-values) derived from bivariable analyses, using Pearson’s chi-square test for categorical variables and the two-sided Mann-Whitney U test for continuous variables. The dashed red line indicates the statistical significance threshold (p = 0.05), and the dotted orange line indicates p = 0.01. Colors identify variable categories: follow-up imaging (red), follow-up hemodynamics (orange), follow-up biomarkers (gold), baseline clinical variables (blue), baseline risk scores (green), baseline biomarkers (purple), composite endpoint components (dark red), and treatment variables (gray). Longer bars indicate stronger statistical evidence of an association with the primary composite endpoint. CT: computed tomography; NT-proBNP: N-terminal pro-B-type natriuretic peptide; PESI: Pulmonary Embolism Severity Index; PMT: percutaneous mechanical thrombectomy; RV: right ventricle; SBP: systolic blood pressure; sPESI: simplified PESI.

### Rationale for the composite endpoint and clinical significance of shock/hypotension

The composite endpoint, comprising all-cause mortality, invasive mechanical ventilation, ECMO, and shock or hypotension requiring vasopressor support, was prespecified because these events represent a clinical continuum of RV failure and progressive circulatory decompensation.^15-17^ In the specific context of intermediate-high- and high-risk PE, where patients were considered for reperfusion because of the severity of their hemodynamic impairment, persistent or recurrent shock requiring vasopressor support should not be regarded as a minor outcome. Rather, it reflects ongoing pulmonary vascular obstruction, failure of RV recovery despite treatment, and an established surrogate for short-term mortality.^1,5^ This conceptual framework is consistent with the PEITHO trial, in which hemodynamic decompensation was the principal contributor to the composite endpoint, and with the 2026 AHA/ACC guideline on PE, which identifies failure to maintain hemodynamic stability as a defining feature of treatment failure in high-risk PE.^5,14^

Although the individual components of the composite endpoint differ in clinical severity, they all indicate failure to achieve cardiovascular stabilization following the diagnosis and treatment of acute PE. In our cohort, persistent shock requiring vasopressor support was the most frequent component, whereas ECMO and invasive mechanical ventilation occurred less often and identified patients with more advanced cardiopulmonary compromise. Accordingly, the observed associations should be interpreted as predictors of overall in-hospital clinical deterioration rather than mortality alone. Because persistent shock was included as a component of the primary composite endpoint, we also developed a longitudinal model that excluded cardiogenic shock at admission. The consistency of the findings across both multivariable models supports the robustness of the observed associations.

### Longitudinal assessment of right ventricular dysfunction

The superior discriminative performance of persistent RV dysfunction on follow-up CTPA (χ^2^ = 15.48), compared with baseline CTPA assessment (p = 0.302), supports the hypothesis that serial evaluation of RV function provides incremental prognostic information beyond the initial assessment. This finding is consistent with meta-analytic evidence demonstrating an approximately 2.5-fold increase in mortality among patients with a baseline RV/LV ratio ≥ 1.0.^6^ The stronger association observed for follow-up CTPA in our cohort may reflect the lower interobserver variability of CTPA-based measurements, the greater standardization of the RV/LV ratio, and the ability of serial imaging to identify failure of RV recovery over time.

RV dysfunction assessed by echocardiography was also significantly associated with the primary composite endpoint (χ^2^ = 11.18, p < 0.001).^7^ The lower chi-square statistic compared with follow-up CTPA may be attributable to the greater operator dependence of echocardiography and to variability in its availability across participating centers.^24-27^

Although follow-up CTPA demonstrated the strongest prognostic association in this cohort, this finding should be interpreted within the context of routine clinical practice. Repeat CTPA was not mandated by the study protocol and was frequently performed to investigate persistent symptoms, assess residual RV dysfunction, or clarify the response to treatment. Consequently, patients who underwent follow-up imaging likely represented a clinically selected subgroup with a higher probability of residual disease and adverse outcomes. Therefore, our results should not be interpreted as evidence that serial CTPA is superior to echocardiographic follow-up. Rather, they support the broader concept that persistent RV dysfunction detected during hospitalization, regardless of the imaging modality used, carries important prognostic information.

Collectively, these findings reinforce the concept that risk stratification in acute PE should not be regarded as a single assessment performed at hospital admission. Instead, patient risk appears to evolve according to the trajectory of RV recovery and hemodynamic stabilization. Initial risk classification defines baseline disease severity, whereas serial assessment identifies patients who remain at increased risk despite receiving appropriate treatment.

### Model calibration

The Hosmer-Lemeshow goodness-of-fit test for Model 2 yielded a χ^2^ value of 7.00 (df = 6, p = 0.321), indicating satisfactory calibration within the study cohort (Figure 4 and Table 8).^21^ This result supports the reliability of the model’s predicted risks across the observed range of outcome probabilities. Nevertheless, external validation in an independent cohort is required before the model can be considered for routine clinical application.^18^

**Table 8:** – Internal validation (bootstrap)

Model performance metric	Value
**Apparent model performance**	
McFadden’s pseudo-R2	0.381
Hosmer-Lemeshow χ^2^ (p-value)	7 (0.321)
Apparent AUC-ROC	0.874
**Bootstrap internal validation (1,000 resamples)**	
Optimism-adjusted AUC-ROC	0.852
Optimism-adjusted calibration slope	0.860
Optimism-adjusted calibration intercept	0.040

AUC-ROC: area under the receiver operating characteristic curve.

### Hemodynamic evolution and systolic blood pressure

Follow-up SBP emerged as a continuous, statistically significant, independent predictor of the primary composite endpoint (Mann-Whitney *U* test, p < 0.001; multivariable OR 0.90 per mmHg). Each 1 mmHg reduction in follow-up SBP may reflect progression from compensated to decompensated RV failure, consistent with the pathophysiology of acute cor pulmonale, in which even modest reductions in SBP among initially normotensive patients may signal progressive RV-LV uncoupling and impending circulatory collapse.^25^ Persistent RV dysfunction combined with lower follow-up SBP therefore identifies a phenotype of incomplete physiological recovery. This dynamic assessment complements, rather than replaces, established admission-based risk stratification systems.

### Informative missing data and their implications

The missing-data analysis demonstrated that patients without follow-up CTPA had substantially greater disease severity at admission, including an approximately fourfold higher median NT-proBNP concentration (4,100 vs. 924 pg/mL, p = 0.001) and a median PESI score approximately 25 points higher (125 vs. 100, p < 0.001). This pattern is consistent with a MNAR mechanism, as originally described by Rubin,^22^ indicating that complete-case analysis in Model 2 may have produced biased estimates, although the direction and magnitude of this bias remain uncertain.^22,23^

Importantly, the observed MNAR pattern may have influenced both effect size estimation and patient selection. Patients who were unable to undergo follow-up CTPA because of early clinical deterioration may have represented a subgroup with persistent RV dysfunction and poorer outcomes that was not captured in the complete-case analysis. Conversely, patients who experienced rapid clinical improvement may also have been less likely to undergo repeat imaging because additional CTPA evaluation was considered unnecessary. Consequently, the observed association between persistent RV dysfunction and adverse outcomes should be interpreted with appropriate caution. Furthermore, because repeat CTPA was performed at the discretion of the treating physicians, the decision to obtain follow-up imaging may have been influenced by the patient’s evolving clinical status or echocardiographic findings, introducing an additional source of post-baseline selection bias.

Future studies should incorporate protocol-mandated serial imaging irrespective of clinical trajectory or apply statistical approaches specifically designed to address informative missing data, including multiple imputation and sensitivity analyses appropriate for MNAR mechanisms.

### Reperfusion strategies and confounding by indication

The management of intermediate-high- and high-risk PE has evolved substantially with the increasing adoption of catheter-based reperfusion therapies.^28-33^ The PEITHO trial^5^ demonstrated that systemic fibrinolysis reduced the composite endpoint of death or hemodynamic decompensation at 7 days (OR 0.44, p = 0.02), although this benefit was accompanied by a higher risk of major bleeding. Subsequently, the FLARE study^12^ showed significant improvement in the RV/LV ratio following mechanical thrombectomy with the FlowTriever system in patients with intermediate-risk PE, and the FLAME study^13^ reported favorable outcomes in selected patients with high-risk PE treated with large-bore mechanical thrombectomy.

More recent randomized evidence from the PEERLESS trial^34^ further supports the expanding role of catheter-based reperfusion, demonstrating improved clinical outcomes with mechanical thrombectomy compared with catheter-directed thrombolysis in selected patients with intermediate-risk PE. Likewise, the STORM-PE trial^35^ has provided additional prospective evidence supporting the efficacy and safety of contemporary catheter-based reperfusion strategies.

Collectively, these studies suggest that successful reperfusion should not be considered the final stage of risk assessment. Instead, the growing use of advanced reperfusion therapies underscores the importance of continued evaluation of RV recovery and hemodynamic stabilization during hospitalization. The findings of the RED-EP registry support a dynamic risk stratification paradigm in which serial reassessment of RV function may identify patients who remain at increased risk despite apparently successful reperfusion.

This concept is further supported by recommendations from the PERT Consortium,^32^ which emphasize longitudinal clinical and imaging follow-up after acute PE, particularly in patients with persistent symptoms, residual RV dysfunction, or complex clinical courses.

### Study limitations

This study has several limitations. First, the relatively small sample size (n = 151), particularly the limited number of patients with complete longitudinal CTPA data (n = 60), restricted the complexity of the multivariable analyses and limited statistical power. Although model performance was initially assessed using apparent estimates, bootstrap resampling with 1,000 iterations yielded optimism-adjusted performance metrics that supported the stability and internal validity of the longitudinal model.^18^

Second, the missing-data analysis demonstrated an informative MNAR mechanism, with patients lacking follow-up CTPA generally presenting with greater disease severity at admission. Consequently, complete-case analysis may have produced biased estimates of uncertain direction and magnitude.^22,23^

Third, follow-up CTPA was not mandated by the study protocol and was more likely to be performed in patients with persistent symptoms, incomplete clinical recovery, or ongoing echocardiographic abnormalities. Therefore, the stronger prognostic performance observed for serial CTPA may partially reflect clinical selection rather than an intrinsic advantage of the imaging modality itself.

Finally, therapeutic heterogeneity across participating centers, together with changes in treatment practices over the study period, limits the interpretation and generalizability of comparisons among reperfusion strategies. In addition, analyses according to thrombectomy device subtype were constrained by the small number of patients in each subgroup.

Accordingly, these findings should be regarded as hypothesis-generating and require confirmation in larger prospective studies incorporating protocol-mandated serial imaging and external validation.

## Conclusions

Reassessment of established risk markers during hospitalization may help identify patients who remain at increased risk despite initial risk classification and treatment. These findings support the concept of dynamic risk stratification in acute PE, suggesting that the trajectory of RV recovery and hemodynamic stabilization provides clinically meaningful prognostic information beyond the initial assessment. Further prospective studies are warranted to establish the optimal approach to longitudinal risk assessment and to validate the role of serial evaluation of RV function in routine clinical practice.
